# An assessment of CO_2_ and CH_4_ emissions in a tropical river: from the Kenyir Reservoir to the estuary

**DOI:** 10.7717/peerj.19929

**Published:** 2025-09-03

**Authors:** Daryl Jia Jun Lee, Siti Farhain Mohd Ludin, Wei Wen Wong, Liyang Zhan, Seng Chee Poh

**Affiliations:** 1 Faculty of Science and Marine Environment, Universiti Malaysia Terengganu, Kuala Nerus, Terengganu, Malaysia; 2 Water Studies, School of Chemistry, Monash University, Clayton, Victoria, Australia; 3 Key Laboratory of Global Change and Marine-Atmospheric Chemistry, Third Institute of Oceanograhy, Ministry of Natural Resources, Xiamen, Fujian, China; 4 Institute of Oceanography and Environment, Universiti Malaysia Terengganu, Kuala Nerus, Terengganu, Malaysia

**Keywords:** Greenhouse gas emissions, Hydropower dam, Tropical reservoir, River damming, Tropical river

## Abstract

This study investigates the spatial and seasonal variations in CO_2_ and CH_4_ emissions from the Kenyir hydropower reservoir and its downstream Terengganu River system in Malaysia. Understanding these variations is crucial for assessing whether the greenhouse gas (GHG) budget for this aquatic continuum significantly contributes to global emissions. Malaysia’s distinct monsoonal climate presents a unique opportunity to explore the influence of seasonal hydrological changes on GHG emission dynamics in inland waters. Five sampling campaigns were performed at the reservoir to investigate this, involving three longitudinal surveys from the reservoir downstream along the Terengganu River, and two time-series samplings at the estuary between November 2017 and August 2019. Our findings reveal that GHG emissions from the Kenyir Reservoir are notably higher during the wet season (97 mmol CO_2_ m^−2^ d^−1^ and 2 mmol CH_4_ m^−2^ d^−1^) than during the dry season (54 mmol CO_2_ m^−2^ d^−1^ and 0.8 mmol CH_4_ m^−2^ d^−1^). This increase coincides with increased wind speed and potential surface mixing during the wet season. Despite operating since 1985, the Kenyir Reservoir’s total GHG emissions remain high compared to other global reservoirs, likely due to its tropical location and high organic carbon content. Elevated GHG emissions were recorded along the Terengganu River, near the dam discharge outlets, with gradual reductions observed downstream. Despite the estuary’s smaller surface area, more GHGs are emitted there than in the river. Overall, the Terengganu River catchment emits approximately 572 Gg CO_2_-equivalent annually, with the Kenyir Reservoir accounting for the majority (94%). The river and the estuary contribute 0.5% and 5.5%, respectively. This study highlights the substantial role of tropical hydropower reservoirs and their downstream river networks in the global GHG budget, emphasizing the need for further investigation into the factors influencing GHG dynamics in tropical river systems.

## Introduction

River fragmentation through dam installation to meet the needs for food security (including water), safety (flood protection), and electricity (hydropower) has been extensively implemented in the rapidly growing region of Asia (Barbarossa et al., 2020). Major river basins, such as the Wujiang (Wang et al., 2021), Mekong (Liu et al., 2020), and Amazon (Kemenes, Forsberg & Melack, 2007), have been fragmented by dams. This fragmentation alters river hydrology upstream and downstream, resulting in riparian wetland losses and aggregates large amounts of organic matter, affecting biogeochemical cycles within the reservoir and downstream, which in turn induces greenhouse gas (GHG) emissions. Numerous studies have reported changes in the GHG emission due to dam construction (Maavara et al., 2020; Wang et al., 2018). Large portions of GHG can rapidly escape to the atmosphere due to hydrostatic drops as bottom water passes through turbine’s discharge outlets. Meanwhile, the remaining carbon dioxide (CO_2_) and methane (CH_4_) in solutions after water passes through a dam either diffuse into the atmosphere or undergo complex biogeochemical processes downstream of the dam.

In cascading reservoir systems, such as in the upper Mekong, the potential for CH_4_ production could significantly increase due to the continuous entrapment of sediment organic matter over successive damming events (Liu et al., 2020). Long periods of water retention in deep reservoirs promote the production of CH_4_ and CO_2_ in the bottom layer of reservoirs due to hypoxic conditions and the accumulation of organic carbon (Müller et al., 2019). The discharge of GHG-supersaturated and anoxic water from hydropower dams can significantly deteriorate downstream riverine systems, affecting physical, biological, and chemical water properties (Reis et al., 2020; Winton, Calamita & Wehrli, 2019). There is growing concern about GHG emissions downstream from tropical hydropower dams. Previous studies have shown that GHG emissions in tropical dammed rivers vary significantly, ranging from 100 to 1,000 mmol CO_2_ m^−2^ d^−1^ and 0.5 to 290 mmol CH_4_ m^−2^ d^−1^ (Abril et al., 2005; Kemenes, Forsberg & Melack, 2007; Kemenes, Forsberg & Melack, 2016).

Urban estuaries are often regarded as a net source for CO_2_ and CH_4_, contributing an approximately 0.27 Pg C yr^−1^ to the atmosphere (Laruelle et al., 2013). The upstream regions of urban estuaries are typically supersaturated with GHG and nutrients. At the same time, adjacent oceanic waters are usually undersaturated due to lower terrestrial organic carbon input and higher biological productivity. Freshwater input from dammed rivers increases GHG levels in estuaries, but these levels generally decrease with salinity through dilution with undersaturated ocean water. However, GHG sources and sinks in estuaries are highly variable, influenced by a combination of biogeochemical processes and tidal dynamics, which change spatially and temporally across different water conditions (Borges & Abril, 2011; Geyer, Chant & Houghton, 2008; Pfeiffer-Herbert et al., 2019). Urbanized estuaries are modified with breakwaters and similar structures to protect harbours and shorelines. These alterations change estuarine hydrodynamics, disrupt freshwater discharge, and increase nutrient loadings, potentially causing eutrophication, anoxia, and higher GHG emissions.

In Malaysia, despite a vast renewable freshwater resource (∼580 billion m^3^; Food and Agriculture Organization (FAO), 2021), few studies have examined GHG emissions from aquatic systems. This study examines GHG emissions within a modified aquatic continuum comprising the Kenyir Reservoir and the Terengganu River. Kenyir Reservoir, one of Malaysia’s largest man-made water bodies, was impounded in 1985 without vegetation clearance, leaving behind organic matter that now fuels bottom water GHG production. The downstream Terengganu River, spanning 61.5 km, receives hypolimnion discharge from the reservoir and input from other urbanized tributaries, such as the Berang, Tersat, Telemong, and Nerus Rivers. At the river mouth, the Terengganu River Estuary (TRE), a salt-wedge system modified by a semi-enclosed breakwater experiences altered water mixing dynamics due to restricted freshwater and seawater exchange. This setting, influenced by its coastal location and human interventions, offers a valuable case for studying how engineered modifications affect GHG emissions and water management.

This study conducted multiple spatio-temporal surveys to map CO_2_ and CH_4_ emissions, identify GHG hotspots, and assess the impact of reservoir damming on emission intensity. By integrating these results with global data, we gain valuable insights into how engineered modifications affect water quality and GHG dynamics, as well as the contribution of the Kenyir Reservoir and Terengganu River to global GHG emissions.

## Materials & Methods

### Study site and sampling

**Figure 1:**
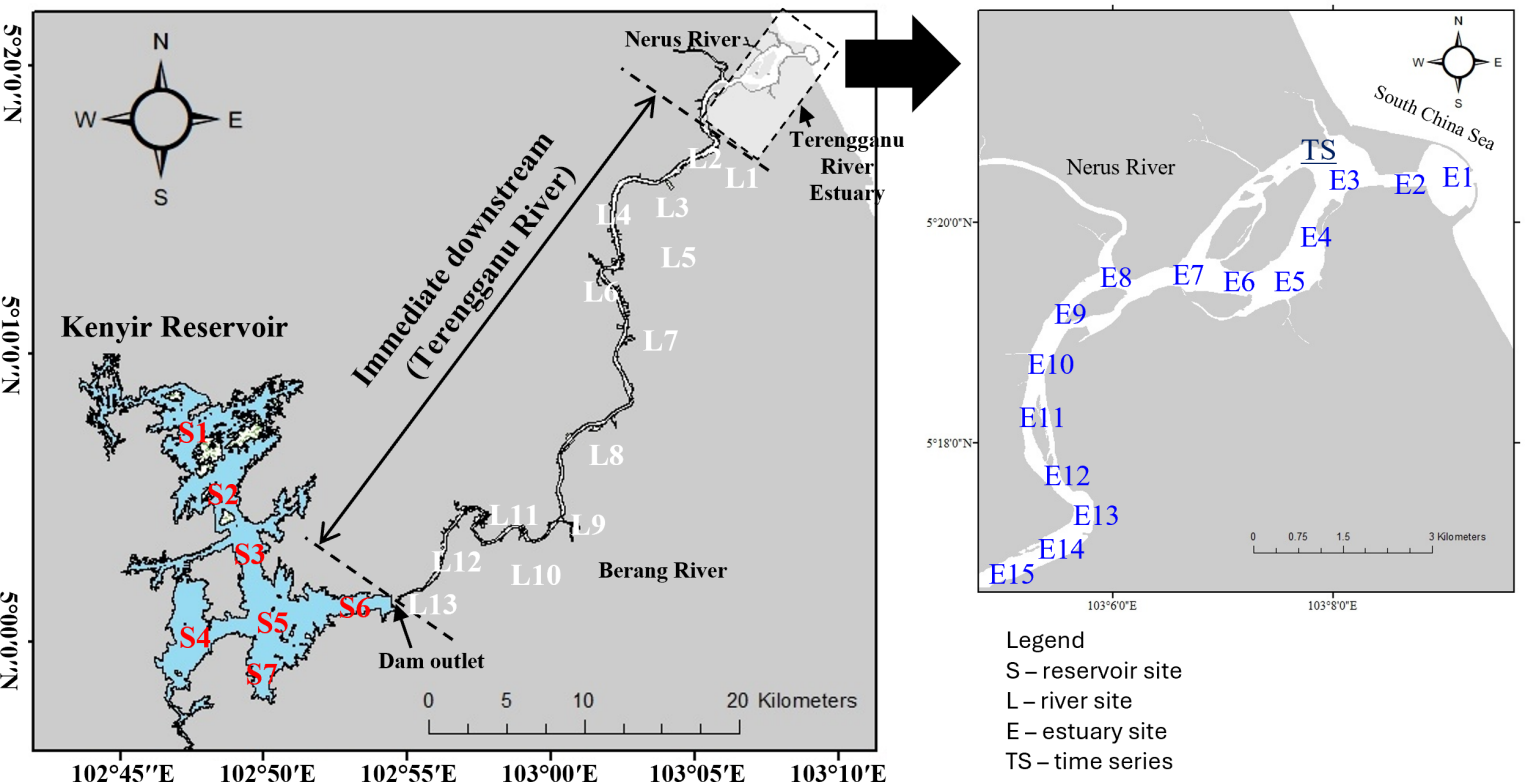
Study area and sampling sites.

Water sampling campaigns were divided into three areas: (i) Kenyir Reservoir (main reservoir only), (ii) Terengganu River (water head and intermediate section, ≈50 km length) and (iii) Terengganu River Estuary (downstream, 15 km length) (Fig. 1). Five sampling campaigns were conducted within two seasons (dry and wet) in seven sampling stations (S1–S7) in Kenyir Reservoir. The reservoir has a maximum depth of 145 m, and the maximum water level drawdown was limited to 10 m; with a minimum dam operating level at 120 m (Rouf et al., 2010). Both dry (March, May, July 2018) and wet (February and November 2018) seasons field sampling campaigns were determined based on local rainfall data obtained from the nearby hydrological station (Sg Gawi station, Kenyir).

Three longitudinal samplings were conducted immediately downstream of the Kenyir Dam under two contrasting river discharge conditions. 13 sampling stations (L1–L13) were chosen depending on land use, logistics and accessibility through jetty and bridges. The sampling campaigns covered ≈50 km in river distance, from the dam outlets to 15 km from the mouth of the Terengganu River. The monthly averaged-discharge rate of the Terengganu River varied from 147 to 224 m^3^ s^−1^ (data obtained from the Department of Irrigation and Drainage Malaysia), showing relatively little variation between wet and dry seasons. The river sampling campaigns were designed to capture different discharge conditions rather than focusing on seasonal differences. Specifically, the March and April 2018 sampling campaigns were carried out during the high-discharge season, with river flows exceeding 150 m^3^ s^−1^. In contrast, the April 2019 campaign was conducted during the low-discharge season when river flows were below 150 m^3^ s^−1^.

The estuarine sampling campaigns were divided into three longitudinal and two time series samplings. The estuarine is a shallow tropical salt-wedge estuary with a depth of 1 to 10 m (average depth: 4.7 m). The longitudinal sampling consists of 15 depth profiling stations (E1–E15) covering a transect distance of 15 km starting from the Terengganu River mouth (Fig. 1). The sampling was conducted during December 2017 (high flow), September (low flow) and December 2018 (high flow). In addition, two 24-hour stationary time series samplings were conducted at different tidal regimes in April 2019 (spring tide) and August 2019 (neap tide). For time series sampling, vertical profile physical water quality measurement was collected every hour, while GHG and nutrients sample collection for surface and bottom depths were collected every two hours.

Data S1 and Table S1 summarise the study sites and sampling activities conducted in the reservoir, river, and estuary during each campaign.

All *in situ* physical water quality measurements (pH, temperature, conductivity, dissolved oxygen, and hydrostatic pressure) were taken with a calibrated YSI 6-Series Multiparameter Water Quality Sonde (YSI 6600, USA). The vertical water column profile was done by lowering the probe until it reached a safe bottom with the assistance of a portable sonar profiler to avoid underwater equipment entanglement with submerged debris such as branches of dead trees.

### Greenhouse gases sampling and analysis

Water samples were taken manually with a 5L Niskin water sampler (General Oceanic, Miami, FL, USA) and carefully transferred *via* tubing into pre-baked 60 mL borosilicate serum bottles to ensure laminar flow and minimize bubble formation. The samples were immediately preserved with saturated mercury chloride (0.05% vol/vol), sealed with butyl rubber stoppers and crimped metal caps. The samples were transported back to the Universiti Malaysia Terengganu chemistry lab for gas chromatography (GC) analysis, following the protocol described by Lee et al. (2022).The GHG samples were headspaced by replacing one-third of the water with helium gas. The samples were then left to equilibrate at room temperature for 30 min, followed by CO_2_ and CH_4_ measurements using a gas chromatograph (Agilent 7980 GC system). Five mL of headspace was injected into the GC using a gas-tight syringe. The GC was equipped with 0.9 m by 1.6 cm o.d. column packed with 80/100 mesh HayeSep Q (Agilent J&W). For CH_4_ analysis, Helium carrier gas was set at the flow rate of 21 mL min^−1^. The flame ionization detector operated under the following conditions: N_2_ makeup gas two mL min^−1^, H_2_ 48 mL min^−1^, air 500 mL min^−1^, and temperature 250 °C. CO_2_ concentration was measured by a thermal conductivity detector operated at the column temperature of 120 °C and filament temperature of 200 °C. The GC column oven was operated at an initial temperature of 50 °C for 4 min, then programmed to 90 °C, and held at this temperature for 6 min. The calibration preparation procedure for GC analysis was according to Lee et al. (2022).

The ebullition fluxes experiment was conducted in the shallow part of the Kenyir Reservoir. Three submersible chambers were placed about 0.5 m below the surface. 60 mL serum bottles prefilled with deionized water were used for gas collection. A tube with a 21G needle was connected from the submersible chamber nozzle to the bottom of the serum bottle (inlet), and another 21G needle near the bottleneck served as the outlet. The outlet tube’s end was placed lower than the nozzle’s depth.

The setup required priming to remove air from the chamber and tubing using a syringe attached to a three-way valve. Priming was complete once the setup was filled with water. The experiment ran for 24 h with samples taken in triplicate. The gas volume collected was determined by the water loss in the serum bottle (Gao et al., 2013); CO_2_ and CH_4_ concentrations were measured using GC as described above. Figure S1 shows the detailed design of the submersible chamber.

### Greenhouse gases diffusive flux across the water–air interface

The diffusive flux of CO_2_ and CH_4_ was obtained by estimating the dissolved GHG concentration of surface water and gas transfer coefficient, *k*_600_ (Ferrón et al., 2007; Wanninkhof, 1992). The *p*CO_2_ (or *p*CH_4_) flux is given in Eq. (1).

(1) \begin{eqnarray*}Gas~Flux={k}_{i}{K}_{i}(pGa{s}_{2water}-pGa{s}_{2atm})\end{eqnarray*}

where *Gas Flux* is the outgassing of CO_2_ or CH_4_ across air-water interface (mmol m^−2^ d^−1^), *k*_*i*_ (cm h^−1^) is the gas transfer velocity coefficient, *K*_*i*_ is the solubility constant of CO_2_ or CH_4_ (mol L^−1^ atm^−1^), *pGas*_*water*_ (µatm) is the partial pressure of CO_2_ or CH_4_ in surface water, and *pGas*_*atm*_ (µatm) is the partial pressure of CO_2_ or CH_4_ in atmosphere.

Gas transfer velocity, *k*_*i*_ is influenced by water temperature and salinity. Following the method specified by Ferrón et al. (2007), *k*_600_ (cm h^−1^) values from each parameterizations was used to calculate *k*_*i*_ for each gas at the recorded temperature and salinity in the field using Eq. (2):

(2) \begin{eqnarray*}{k}_{i}={k}_{600}(S{c}_{i}\div 600)^{n}.\end{eqnarray*}

For the reservoir, we used *n* =  − 2/3 for the wind speed <3.7 m/s and −1/2 for higher wind speed (Wanninkhof, 1992). For rivers and estuaries, where the water-air interface is expected to be turbulent rather than smooth, *n* =  − 1/2 was applied (Guérin et al., 2006; Wanninkhof, 1992). For the Schmidt numbers, *Sc*_*i*_ is the ratio of the kinematic viscosity of water over the diffusivity of the gas (Sc = *ν*/D). The formulation of *Sc*_*i*_ is derived from Wanninkhof (1992) and expressed in Eq. (3):

(3) \begin{eqnarray*}Sc=A-Bt+C{t}^{2}-D{t}^{3}\end{eqnarray*}

where *t* is in degrees Celsius (^∘^C), and A, B, C and D are constant for the coefficients listed in Wanninkhof (1992) (refer to Table S2).

Since this study did not measure *k*_600_, gas fluxes were estimated using parameterizations of *k*_600_ reported in the literature (Table S3). The final flux determination was obtained by averaging all parameterizations applied for the reservoir and estuary study areas. In contrast, for the lake study area, the Crusius & Wanninkhof (2003) (C&W03) parameterization was exclusively used to estimate fluxes. C&W03 parameterization for the lake was used in the Terengganu River due to the absence of accessible water velocity and depth measurements, which are typically required for accurate *k*_600_ estimation in river sections. C&W03, previously applied in a reservoir study, was chosen because it provides the highest transfer velocity (cm h^−^^1^) value, offering a conservative upper limit estimate. This approach ensures a comprehensive and cautious estimation of gas fluxes within the constraints of the available data.

In this study, wind data for the *u*_10_ (m s^−1^) in *k*_600_ was taken from Zippenfenig (2024) global model (Table S4) and applied in the literature *k*_600_. The local wind speed data was extrapolated to wind speed at 10 m (*u*_10_), according to Crusius & Wanninkhof (2003), Eq. (4):

(4) \begin{eqnarray*}{u}_{10}={u}_{z} \left[ 1+ \frac{({C}_{d10})^{1/2}}{K} \ln \nolimits ( \frac{10}{z} ) \right] \end{eqnarray*}

where *z* is the measured wind speed height, *C*_*d*10_ is the drag coefficient at 10 m in height (0.0013; Stauffer, 1980), and *K* is the Von Karman constant (0.41).

The ebullition flux was obtained by using the following Eq. (5) from IHA (2010):

(5) \begin{eqnarray*}\text{Ebullition Flux}~(\mathrm{mg}{\mathrm{m}}^{-2}{\mathrm{d}}^{-1})= \frac{\text{Gas Con}. \left( \mathrm{mg}{\mathrm{m}}^{-3} \right) \times \text{Gas Vol}.\text{Collected}({\mathrm{m}}^{3})}{\text{Funnel Area} \left( {\mathrm{m}}^{2} \right) \times \text{Sampling Interval}(\text{days})} \end{eqnarray*}

where gas con. is the measured GHG (CO_2_ and CH_4_), gas vol. collected is the volume of GHG collected in the serum bottle, and funnel area is the surface area of the inverted chamber (refer to Fig. S1).

The GHG emissions were estimated using a surface area-based approach. Surface areas of the Kenyir Reservoir, the Terengganu River, and its estuary were digitized from satellite imagery. The average GHG concentration was determined from field measurements at multiple locations within each waterbody. The total GHG flux was then calculated using the Eq. (6), where the flux density was derived from *in situ* measurements and expressed in mmol m^−2^ d^−1^.

(6) \begin{eqnarray*}Total~GHGs~Emission~ \left( mmol~{m}^{-2}{d}^{-1} \right) =surface~area~ \left( {m}^{2} \right) \times \text{mmol}~{m}^{-2}{d}^{-1}.\end{eqnarray*}

### Data visualization and statistical analysis

The spatial distribution of GHG and fluxes was mapped using ArcGIS and the Ocean Data Viewer (ODV, AWI) software. A variable marker size approach was employed to display individual data points. Instead of simply showing coloured dots at sample points for visualizing heatmap continuity, the weighted-average gridding spatial interpolation technique available in ODV was used (Schlitzer, 2002). The interpolation X and Y scale-lengths were minimized to prevent overlapping coverage between data points and balance the data structure and smoothness preservation.

The dataset was characterized using descriptive statistics (minimum, maximum, average, median, and standard deviation) performed with SPSS statistical software, and seasonal trends were averaged for comparison. Since the Shapiro–Wilk test (*p* < 0.05) indicated that the data did not follow a normal distribution, non-parametric methods were used. Specifically, Spearman’s rho was applied to assess correlations, and the Kruskal–Wallis test was employed to compare means across stations, depths, and periods.

## Results

### Water physical properties

The reservoir was thermally stratified during dry and wet periods (Fig. S2). Surface water temperature ranged from 28.5 °C to 30.5 °C across the study sites. The average temperature difference between the surface and bottom water was significant (*p* < 0.05, ±5.0 °C). Under stratification conditions, the reservoir maintained a permanent hypoxic hypolimnion. The vertical distribution of oxygen saturation generally became hypoxic after 40 m depth, but several second oxyclines were observed between 20 and 40 m water depth at several shallow sites.

Figure S2 shows that the oxygen saturation levels (DO) differed significantly among the upstream (L1–L4), middle (L5–L8), and downstream (L9–L13) sections of the Terengganu River (Kruskal–Wallis test, *p* < 0.05). Notably, the DO level immediately downstream of the dam (L1, 1 km) was consistently lower (41 DO%) than other parts of the river (72 DO%). The spatial extent of low DO concentrations in the upstream sections (L1–L4) was directly associated with dam outflow averaged-discharge rates. During the high discharge periods in March 2018 and April 2018, with dam outflow averaged-discharge rates recorded at 163 and 154 m^3^ s^−1^, respectively, low DO levels (<50 DO%) extended 11–12 km downstream from the dam (Fig. S2). In contrast, the low flow period in April 2019, with an outflow averaged-discharge rate of 124 m^3^ s^−1^, resulted in a considerably reduced spatial extent of low DO, confining the affected area to within one km of the dam outlet.

The longitudinal and vertical profiling of salinity at the estuary indicates that the area is a partially mixed estuary, with tidal amplitudes averaging between 0.8 and 2.9 m. Figure S2 shows that the maximum seawater intrusion occurred approximately eight km from the estuary’s mouth (E8–E15). The shorter saltwater intrusion observed in this study could be attributed to the presence of a breakwater outside the estuary inlet. The Terengganu estuary inlet is protected by a semi-circular coastal breakwater, which has only a small opening to the sea, thereby limiting freshwater outflows.

During September 2018 sampling (low flow), DO levels in the estuary were typically lower near the substrate and higher closer to the water’s surface. In contrast, DO levels were homogeneous throughout the water column during the December 2017 sampling (high flow). The variation in DO concentrations between the two sampling campaigns may be due to seasonal differences in river discharge rates. Although river discharge data were unavailable for the December 2017 sampling campaign, the water level in December 2017 (7.8 m) was higher than in September 2018 (7.3 m). River water level is directly proportional to flow velocity (Mohd Saupi et al., 2018). Consequently, the estuary’s dissolved oxygen levels were often not vertically stratified during high discharge events, as strong river flows tend to homogenize the water column and disrupt any oxyclines. In addition, the DO concentration in the estuary during December 2017 was much lower than during September 2018.

### GHG concentrations and fluxes

The CO_2_ and CH_4_ concentrations in the Kenyir Reservoir were supersaturated, with the CO_2_ ranging from 96 to 616 µM, and CH_4_ from 0.1 to 127 µM. Seasonally, CO_2_ concentrations showed significant differences (Kruskal–Wallis test *p* < 0.05), while CH_4_ concentrations did not show differences within sampling months (Kruskal–Wallis test, *p* > 0.05). There was a statistically significant difference in CO_2_ and CH_4_ concentrations between the sampling sites (Kruskal–Wallis test *p* < 0.05, Fig. S3).

**Figure 2:**
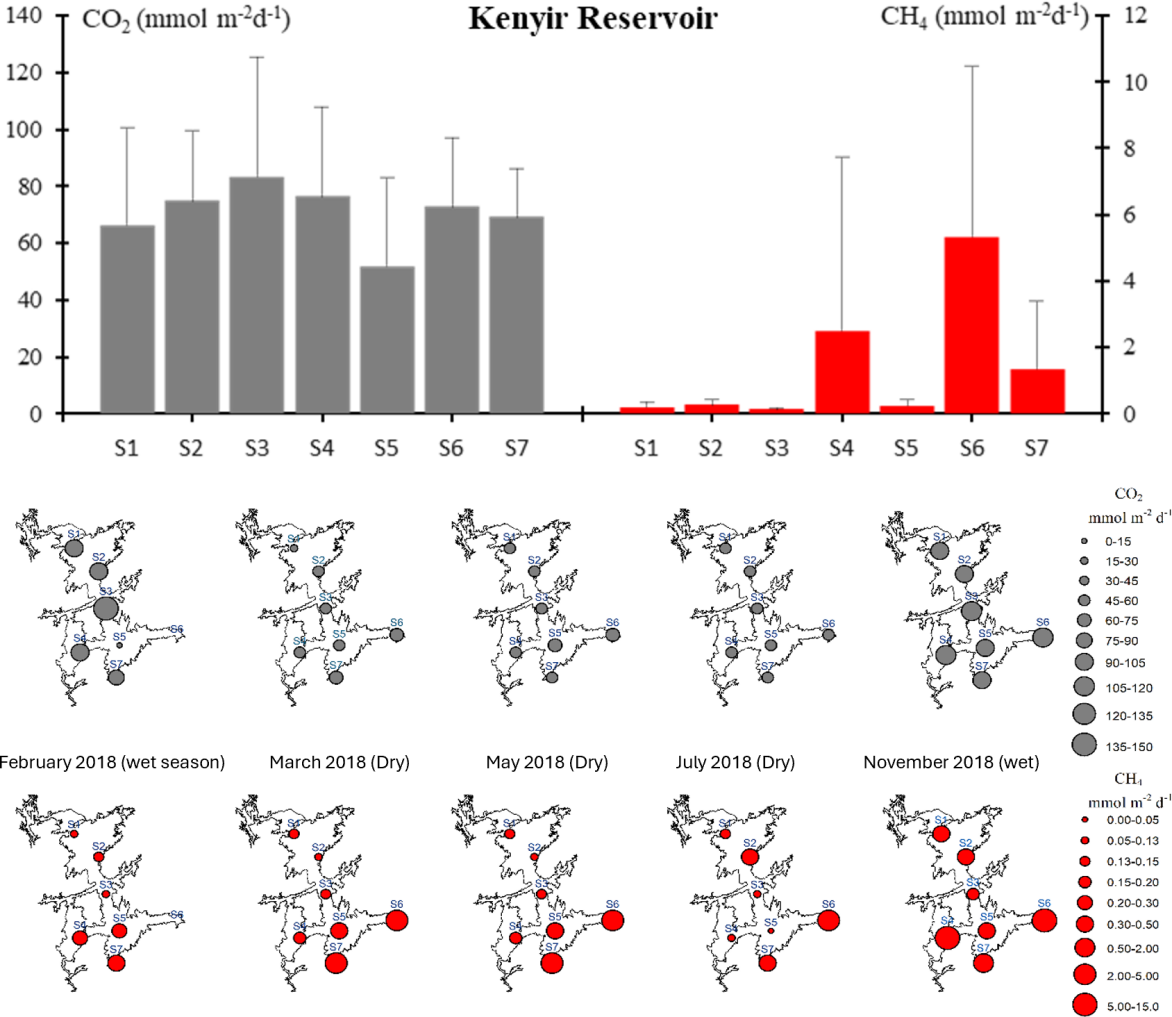
The CO_2_ and CH_4_ emission fluxes pattern in Kenyir Reservoir. Top panel: Averaged CO_2_ and CH_4_ emission fluxes at each sampling site. Middle and bottom panels: Temporal and spatial variations of CO_2_ and CH_4_ emission fluxes across Kenyir Reservoir, respectively.

However, CO_2_ and CH_4_ fluxes in the Kenyir Reservoir did not show significant spatial variations (Kruskal–Wallis test, *p* > 0.05, Fig. S3). During the dry period, CO_2_ fluxes for the Kenyir Reservoir (S1-S7) ranged from 26 to 62 mmol m^−2^ d^−1^, and CH_4_ fluxes ranged from 0.02 to 5.0 mmol m^−2^ d^−1^. During the wet period, CO_2_ fluxes increased significantly compared to the dry period (Kruskal–Wallis test *p* < 0.05), ranging from 5.6 to 143 mmol m^−2^ d^−1^, while CH_4_ fluxes did not show significant seasonal variation (Kruskal–Wallis test *p* > 0.05), ranging from 0.1 to 12 mmol m^−2^ d^−1^. Higher CO_2_ and CH_4_ emissions were detected in the surface water of S3, S4, S6, and S7 (Fig. 2).

Ebullition fluxes during the dry period were significantly higher (Kruskal–Wallis test, *p* < 0.05), with CH_4_ reaching 3.79 × 10^−2^ mmol m^−2^ d^−1^ and CO_2_: 1.56 × 10^−2^ mmol m^−2^ d^−1^, respectively. These rates were at least an order of magnitude higher than those observed during the wet period (CH_4_: 3.94 × 10^−8^ mmol m^−2^ d^−1^ and CO_2_: 1.66 × 10^−4^ mmol m^−2^ d^−1^).

**Figure 3:**
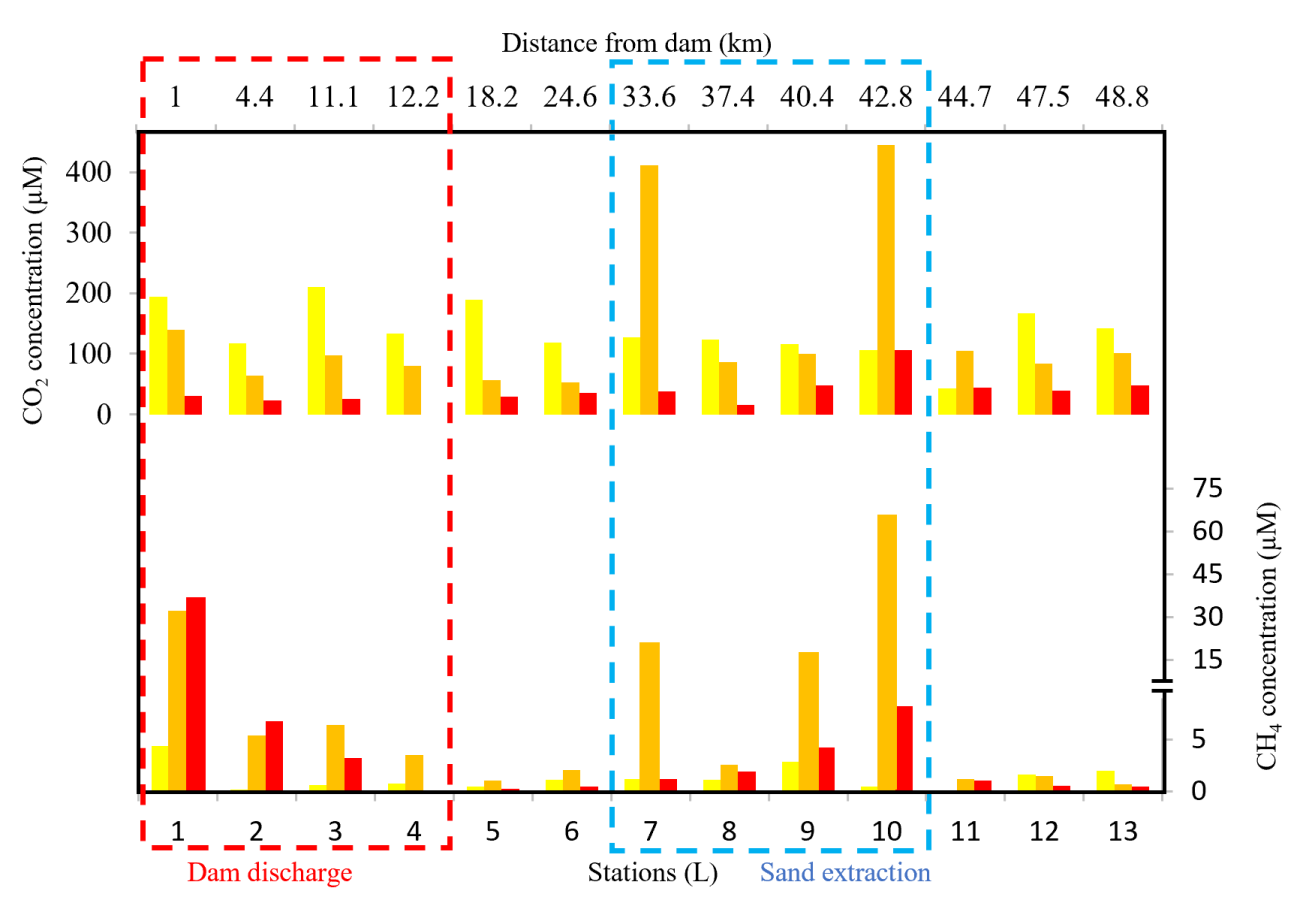
Longitudinal distribution of CO_2_ and CH_4_ concentration in Terengganu River. Yellow, March 2018; orange, April 2018; red, April 2019.

Averaged CH_4_ concentrations downstream of Kenyir Reservoir (Fig. 3) were significantly higher during the high discharge period (9.1 ± 15 µM, range: 0.2 to 65 µM) than during the low discharge period (1.3 ± 1.2 µM, range: 0.001 to 4.4 µM, Kruskal–Wallis test *p* < 0.05). High CH_4_ and CO_2_ concentration anomalies were observed in the upstream (L1–L4) and the middle segment (L7–L10) of the Terengganu River, indicating a potential hotspot for GHG emission.

**Figure 4:**
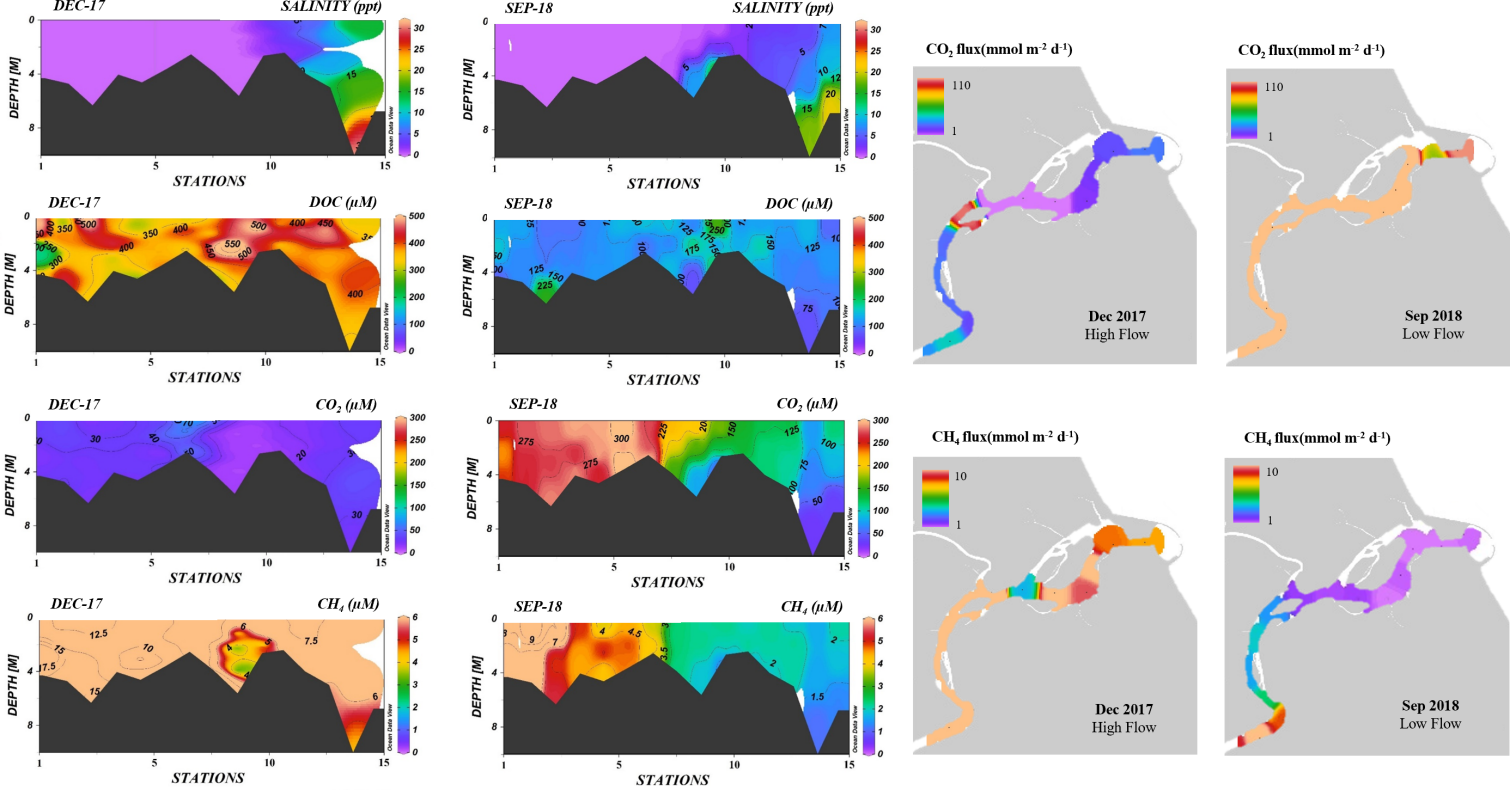
Longitudinal profiles of CO_2_ and CH_4_ concentrations and fluxes along the Terengganu River Estuary during high (Dec 2017) and low flow events (Sept 2018).

In the Terengganu River Estuary, CO_2_ and CH_4_ concentrations across all sites varied between December 2017 (high flow) and September 2018 (low flow, Fig. 4). The CH_4_ concentrations ranged from 0.2 to 20 µM during high flow, and between 1.1 to 10 µM during low flow. CO_2_ concentrations ranged from 7 to 307 µM during high flow and 27 to 317 µM during low flow.

GHG flux measurements in estuary during high flow showed that CH_4_ fluxes averaged 13 ± 5.0 mmol m^2^ d^−1^ (range: 4.3 to 21 mmol m^2^ d^−1^), while CO_2_ fluxes averaged 20 ± 30 mmol m^2^ d^−1^, (range: −11 to 112 mmol m^2^ d^−1^, as shown in Fig. 4). In contrast, CH_4_ fluxes remained relatively stable during low flow, averaging 4.1 ± 2.8 mmol m^2^ d^−1^ (range: 1.6 to 10 mmol m^2^ d^−1^). However, CO_2_ fluxes were significantly higher (Kruskal–Wallis test *P* < 0.05), with an average of 232 ± 95 mmol m^2^ d^−^^1^ (range: 70 to 350 mmol m^2^ d^−1^).

Time-series measurements during neap and spring tides revealed notable differences in GHG concentrations and fluxes. During neap tide, CH_4_ concentrations averaged 1.5 ± 0.6 µM, (range: 0.4 to 2.6 µM), while CO_2_ concentrations averaged 56 ± 30 µM (range: from 20 to 109 µM). In contrast, CH_4_ concentrations were lower during spring tide, averaging 0.6 ± 0.4 µM (range: below detection limit to 1.4 µM), and CO_2_ concentrations were slightly higher, averaging 61 ± 38 µM (range: 24 to 126 µM).

**Figure 5:**
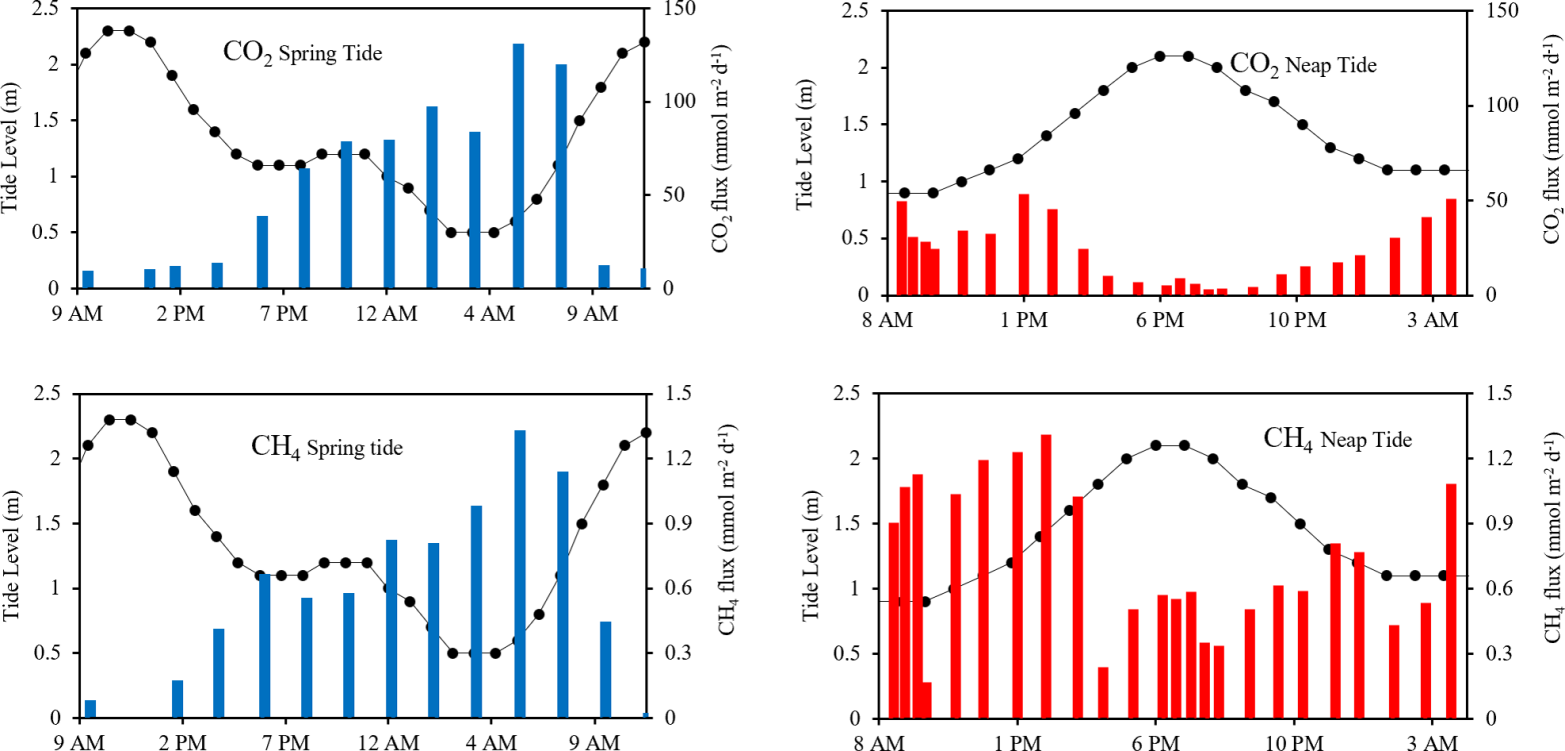
Diurnal variation of CO_2_ and CH_4_ fluxes in Terengganu River Estuary. Black coloured line represents tide height in m; coloured bars represent the flux in mmol m^−2^ d^−1^.

Over a 24-hour sampling period, GHG fluxes during neap tide showed that CH_4_ averaged 0.7 ± 0.3 mmol m^2^ d^−1^ (range: 0.2 to 1.3 mmol m^2^ d^−1^, Fig. 5), while CO_2_ fluxes averaged 23 ± 16 mmol m^2^ d^−1^ (range: 3.2 to 53 mmol m^2^ d^−1^) During spring tide, the CH_4_ fluxes averaged 0.6 ± 0.4 mmol m^2^ d^−^^1^ (range: −0.09 to 1.3 mmol m^2^ d^−1^), whereas CO_2_ fluxes increased, averaging 55 ± 44 mmol m^2^ d^−1^ (range: 10 to 131 mmol m^2^ d^−1^).

## Discussion

### Greenhouse emissions from Kenyir Reservoir

Temperature and dissolved oxygen profiles indicate that the Kenyir Reservoir is a meromictic system with stable thermal stratification year-round (Fig. S2). This thermal stratification drives oxygen stratification, isolating bottom waters from atmospheric exchange and resulting in anoxic conditions that enhance methane production through methanogenesis (Davidson, Audet & Jeppesen, 2018). Under these low-oxygen conditions, organic matter decomposition also contributes to elevated carbon dioxide levels. As a result, oxygen stratification enhances both CH_4_ and CO_2_ production in deeper waters (Fig. S3).

Statistically, the average CO_2_ emission rate across all sites was significantly higher during the wet season (Kruskal–Wallis test *p* < 0.05). This increase may be attributed to stronger winds. In this study, monthly average wind speed in the Kenyir Reservoir was 1.3 times higher during the wet season than in the dry season (Table S4). This finding aligns with the findings of Sawakuchi et al. (2017) that windier conditions promote surface water mixing and enhance the diffusion of dissolved GHG into the atmosphere.

Ebullition was observed in the shallow southern region near S7 (Fig. S2), likely due to shallow water. Previous studies (Bastviken et al., 2004; Gerardo-Nieto et al., 2017; West, Creamer & Jones, 2016; DelSontro et al., 2010) have shown that ebullition is more common in littoral zones. In the Kenyir Reservoir, active ebullition occurred near to S7, where water depths were less than 40 m.

The overall water level increase in Kenyir Reservoir during the wet season was associated with a lower ebullition rate, as higher hydrostatic pressure suppresses gas bubble formation, similar to the observation of Ostrovsky et al. (2008) in Lake Kinneret. We assume that GHG production from the bottom sediments remains consistent year-round due to the persistent anoxic conditions at the reservoir bottom (Fig. S4). However, intense wind forcing during the wet season (Table S4) enhances internal lake motion, disrupting the ascent of GHG bubbles. McGinnis et al. (2006) demonstrated that increased horizontal currents break larger bubbles into smaller ones, increasing the total surface area available for bubble redissolution before they reach the surface. Consequently, these processes result in fewer GHG bubbles captured by submersible trapping funnels in S7 during the wet season.

Table 1 presents the CO_2_ and CH_4_ emissions from 39 freshwater reservoirs and lakes latitudinally from north to south. Kenyir Reservoir ranks 9th and 8th for CH_4_ and CO_2_ among the global freshwater GHG emissions references in Table 1. The complied data showed that tropical reservoirs release more GHG than boreal, temperate, and subtropical reservoirs. For instance, Tucurui Reservoir in Brazil measured 0–180 mmol CH_4_ m^−2^ d^−1^ and 30–3,242 mmol CO_2_ m^−2^ d^−1^ in 1993 (Dos Santos et al., 2006). Curuá-Una reservoir measured CH_4_ emissions of 0.1–7.0 mmol m^−2^ d^−1^ and CO_2_ emissions of 0.6–1,612 mmol m^−2^ d^−1^ in 2017 (Paranaíba et al., 2021), which were nearly ten times higher than Lake Allatoona, USA, in the sub-tropical reservoir that recorded 11.7 mmol CH_4_ m^−2^ d^−1^ and 54.8 mmol CO_2_ m^−2^ d^−1^ in 2012 (Bevelhimer et al., 2016).

**Table 1:** Comparison of Kenyir Reservoir CH_4_ and CO_2_ emissions with global Reservoirs.

References	Region	Reservoir	Country	Latitude	Age during measurement year	Surface area km^2^	Diffusive flux CH_4_ mmol m^−2^ d^−1^	Diffusive flux CO_2_ mmol m^−2^ d^−1^	C emission Gg C yr^−1^
Huttunen et al. (2004)	Temperate	Reservoir Lokka	Finland	68°N	28	216-417	0.33–7.4	11–73	123
Huttunen et al. (2004)	Temperate	Reservoir Porttipahta	Finland	68°N	25	34–214	0.16–0.3	20–52	83.7
Roehm & Tremblay (2006)	Temperate	La Grande-2	Canada	54°N	26	2815	0–0.16	15 (1–148)	845.6
Roehm & Tremblay (2006)	Temperate	La Grande-3	Canada	54°N	21	2,536	9–34	325.9
Martinez-Cruz et al. (2020)	Temperate	Lake Dagow	Germany	53°N		0.24	2.3	6	0.02
Martinez-Cruz et al. (2020)	Temperate	Lake Stechlin	Germany	53°N	55	4.25	0.09	−5.5	−0.4
Brothers, Prairie & delGiorgio (2012)	Temperate	Eastmain-1	Canada	52°N	2	603	1.4	13.6
Descloux et al. (2017)	Temperate	Eguzon Reservoir	France	47°N	85	2.7	0.03–5.6		0.02
Demarty et al. (2009)	Temperate	Rivière-des-Prairies	Canada	46°N	79	42.3	0–0.4	15 (-5–213)	10.2
Samiotis et al. (2018)	Temperate	Polyfytos Reservoir	Greece	40°N	4	21.9	0–6.7	4.1–45.1	6.9
Samiotis et al. (2018)	Temperate	Ilarion Dam	Greece	40°N	42	74	0–11.6	1.1–41	25.6
Beaulieu et al. (2020)	Subtropical	Acton Lake	USA	40°N	59	2.4	9.3–18.3		0.2
McClure et al. (2018)	Subtropical	Falling Creek Reservoir	USA	38°N	117	0.119	0.7	−4.6–135	0.1
Bevelhimer et al. (2016)	Subtropical	Watts Bar Lake	USA	36°N	70	176	0.5	62.7	177.8
Bevelhimer et al. (2016)	Subtropical	Guntersville Lake	USA	36°N	73	279	1.3	40.8	185.0
Bevelhimer et al. (2016)	Subtropical	Fontana Lake	USA	36°N	68	43	0.4	22.6	15.7
Bevelhimer et al. (2016)	Subtropical	Hartwell Lake	USA	35°N	50	226	1.4	26.5	98.1
Bevelhimer et al. (2016)	Subtropical	Lake Allatoona	USA	34°N	63	49	11.7	54.8	46.5
Liu et al. (2016)	Subtropical	Ross Barnett Reservoir	USA	33°N	45	134		26.8	57.6
Chen et al. (2011)	Subtropical	Three Gorges Reservoir	China	31°N	5	1,080	0.34 ± 0.3	76 ± 10.8 (76–213)	1327.6
Xing et al. (2005)	Subtropical	Lake Donghu	China	31°N		19.03	1.5 ± 1.2	7.6 ± 3.6	2.5
Chanudet et al. (2011)	Tropical	Nam Ngum	Laos	19°N	39	350	0.1–0.6	−21.2 to −2.7	112.0
Deshmukh (2013)	Tropical	Nam Theun 2 Reservoir	Laos	18°N	3	450	0–157	68 ± 51	500.8
Chanudet et al. (2011)	Tropical	Nam Leuk	Laos	18°N	11	13	0.8–12	−11–38	6.6
Prasad et al. (2013)	Tropical	Dowleiswaram dam	India	17°N	24	70		10–473	47.2
Guérin et al. (2006)	Tropical	Petit-Saut Reservoir	France	5°N	9	365	4.6 ± 4.9 (0.1–7.7)	119 ± 98 (102–133)	708
Soued & Prairie (2020)	Tropical	Batang Ai Reservoir	Malaysia	1°N	33	68.4	0.03–3.7	−31–80	8.7
Guérin et al. (2006)	Tropical	Balbina	Brazil	2°S	16	2,360	2.1 ± 3 (0.31–21)	76 ± 46	2913
Paranaíba et al. (2021)	Tropical	Curuá-Una	Brazil	3°S	40	72	0.08–7	3.4–1612	107
Dos Santos et al. (2006)	Tropical	Tucurui	Brazil	4°S	9	2,430	0.002–180	10.4–3243	7606
Almeida et al. (2016)	Tropical	Ecological Station of Serido	Brazil	7°S	70	0.2	11.8 ± 4.1	0.04
Macklin et al. (2018)	Tropical	Palasari Reservoir	Indonesia	8°S	27	1	372	3.5
Guérin et al. (2006)	Tropical	Samuel	Brazil	9°S	17	559	5.0 ± 5.9	976 ± 1213	8,780
Teodoru et al. (2015)	Tropical	Cahora Bassa	Africa	16°S	39	2,675	0.08	−8.1	−347
Teodoru et al. (2015)	Tropical	Itezhi Tezhi	Africa	16°S	35	365	1.6	16.7	101
Paranaíba et al. (2021)	Tropical	Furnas Dam	Brazil	21°S	54	1,342	0.001–21.5	−36–90	117
Paranaíba et al. (2021)	Tropical	Funil Reservoir	Brazil	21°S	13	40	0.0002–2.1	−0.05–1.7	0.1
Paranaíba et al. (2021)	Tropical	Chapéu D’Úvas	Brazil	22°S	23	12	0.02–19	−26–32	1.3
This study	Tropical	Kenyir Reservoir	Malaysia	5°N	31	369	0.02–12.5	5.6–143.8	451.9

Tropical reservoirs exhibit higher GHG emissions (0–180 mmol CH_4_ m^−2^ d^−1^ and −36–3,243 mmol CO_2_ m^−2^ d^−1^) than reservoirs in other climates (0–18.3 mmol CH_4_ m^−2^ d^−1^ and −5.5–213 mmol CO_2_ m^−2^ d^−1^). This difference is largely due to greater organic carbon in tropical reservoirs and higher surface runoff, which continuous supplies organic matter, fueling bacterial metabolism and enhancing GHG production (Tranvik et al., 2009). Table 1 also shows higher GHG emissions are commonly observed in young reservoirs (less than 10 years). For example, in the first year after damming, annual diffusive CO_2_ and CH_4_ emissions in reservoirs, such as Nam Theun 2, Nam Leuk (Laos), Petit-Saut (French Guiana), and Tucurui (Brazil), were approximately 1 to 10 times greater than in later years. Despite being more than 30 years old, the Kenyir Reservoir continues to exhibit high CO_2_ and CH_4_ emission than other reservoirs with similar age, such as the Batang Ai Reservoir (Soued & Prairie, 2020) and Reservoir Lokka (Huttunen et al., 2004).

### Greenhouse gas emissions downstream of a dammed river

High GHG concentrations in the Terengganu River were observed near the dam discharge outlets (4.4–36.5 µM CH_4_ and 30.3–194 µM CO_2_), extending up to 12 km downstream (0.7–3.6 µM CH_4_ and 80–133 µM CO_2_) from L1 to L4 (Fig. 3). In these upstream sections, CH_4_ concentrations were negatively correlated with dissolved oxygen (*r* =  − 0.6, *p* < 0.05), suggesting that the elevated CH_4_ levels originated from the hypoxic bottom waters of the reservoir. Consistently high CH_4_ concentrations were recorded near the reservoir’s bottom (mean 621 ± 187 µM, *n* = 20), where oxygen depletion provides favorable conditions for methanogenesis (Conrad, 2020; Müller et al., 2019). From site L1 to L4, immediately downstream of the reservoir outlets, CH_4_ concentration decreased by approximately 80% (Fig. 3). This decline was likely driven by a combination of dilution, atmospheric evasion, and microbial oxidation (DelSontro et al., 2016b; Guérin & Abril, 2007; Kemenes, Forsberg & Melack, 2007). Further downstream at L5 and L6, CH_4_ concentrations dropped below five µM.

The substantial increase in CO_2_ and CH_4_ concentration in the middle segment of the Terengganu River (L7 to L10, Fig. 3) is most likely linked to in-stream sand extraction activities. At least four active in-stream sand extraction operations were observed during the sampling campaign along this river section. These activities involved submersible pumps extracting riverbed materials comprising gravel, sand, silt, and mud, resulting in the mineralization of resuspended particulate organic matter, releasing substantial CO_2_ and CH_4_ into the overlying water column. In addition, the disturbance of riverbed sediments may have promoted the degassing of CH_4_ and CO_2_ from porewater (Marcon et al., 2022). Qin et al. (2020) also reported that in-stream sand extraction disrupts the carbon sequestration potential of riparian areas, significantly reducing CO_2_-fixing microbial communities and leading to increased GHG emissions. Beyond its biogeochemical impacts, sand extraction disturbs sediment supply and transport equilibrium, triggering morphological alteration that causes irreversible changes in the watershed characteristics (Padmalal & Maya, 2014). The resuspension of sediments increases turbidity in the water column (Ashraf et al., 2011). Over time, reduced light penetration limits photosynthesis and oxygen production, promoting the anaerobic condition in the benthic layer, which enhances microbial decomposition of organic matter, further increasing CO_2_ and CH_4_ production.

The Terengganu River is a source of CO_2_ and CH_4_ to the atmosphere with fluxes ranging from 0.7 to 136 mmol CO_2_ m^−2^ d^−1^ and 0.06 to 20 mmol CH_4_ m^−2^ d^−1^, respectively. In-stream sand extraction is an important contributor to the river’s CH_4_,  and CO_2_ flux. Overall, sand extraction activity alone has contributed more than 50% of Terengganu River’s total carbon (CO_2_+CH_4_) emission (Table 2). Downstream discharge water from the dam is the second largest CH_4_ source, contributing 30% of the diffusive flux to the atmosphere.

**Table 2:** Total diffusive CO_2_ and CH_4_ flux in Terengganu River and the estimated contributions from specific anthropogenic activities.

Sampling period	CO_2_ (mmol m^−2^ d^−1^ ) median ± std	Total river emission (Mmol d^−1^ )	Contribution (%)		
			Dam discharge	Sand extraction	Other
Mar-18	7.5 ± 7.1	0.08	8	49	43
Apr-18	26 ± 42	0.43	10	71	18
Apr-19	37 ± 14	0.40	24	32	45
		**Average contribution (%)**	14	51	35
	**CH_4_ (mmol m^−2^ d^−1^)**				
Mar-18	0.5 ± 3.0	0.01	55	38	7
Apr-18	1.0 ± 5.7	0.03	16	79	5
Apr-19	0.3 ± 0.4	0.003	18	47	35
		**Average contribution (%)**	30	55	15

The median concentration of CO_2_ (99 µM) and CH_4_ (1.6 µM) in the Terengganu River was at least 6 and 8,000 times higher than atmospheric levels (∼17 µM CO_2_; ∼0.002 µM CH_4_), respectively. Overall, CO_2_ and CH_4_ levels in the Terengganu River fall within the typical range of global tropical rivers (42–337 µM CO_2_; 0.4–350 µM CH_4_) but are at least an order of magnitude lower than those in dammed rivers and peat-draining rivers (Table 3). In terms of diffusive flux, Terengganu River CO_2_ (0.7 to 136 mmol m^−2^ d^−1^) and CH_4_ (0.06 to 20 mmol m^−2^ d^−1^) fluxes are comparable to those reported for Wohlen Reservoir (DelSontro et al., 2016b), River Tay (Harley, 2013), Tianjin River (Hu et al., 2018), and as well as Kariba Dam (Teodoru et al., 2015). However, they are one to two orders of magnitude lower than fluxes reported for tropical reservoirs and rivers in Brazil (Abril et al., 2005; Guérin et al., 2006; Kemenes, Forsberg & Melack, 2007; Kemenes, Forsberg & Melack, 2011), Indonesia (Wit et al., 2015), and Malaysia (Müller et al., 2015).

**Table 3:** Greenhouse gases diffusive fluxes at the air-water interface of rivers around the world.

Location	River/Reservoir Immediate Downstream (year of measurement)	Dissolved surface GHGs (µmol L^−1^)		Diffusive flux (mmol m^−2^ d^−1^)		References
		CH_4_	CO_2_	CH_4_	CO_2_	
Switzerland	Wohlen Reservoir (2013)	0.1–1.3	–	0.13–3.0	–	DelSontro et al. (2016a)
Scotland	River Tay (2010)	–	–	0.1–1.0	12–58	Harley (2013)
						
China	Tianjin River (2015)	1.4 ± 1.2	38 ± 8.6	1.7 ± 1.6	20 ± 10	Hu et al. (2018)
						
Brazil	Kariba Dam (2013)	<0.1	106	–	–	Teodoru et al. (2015)
	Itezhi Tezhi Dam (2013)	<0.1	42	1.2	13	
	Cabora Bassa Dam (2013)	–	–	–	34	
	Petit-Saut Reservoir (2003)	2.5 ± 2.6	108 ± 63	59	1,003	Abril et al. (2005)
	Petit-Saut Reservoir (2005)	48	311	84 ± 38	802 ± 364	Guérin et al. (2006)
	Samuel Reservoir (2004)	40	337	12 ± 13	1,494 ± 963	
	Balbina Reservoir (2004)	77 ± 7	203 ± 27	114 ± 66	412 ± 95	
	Balbina Reservoir (2005)	0.4-140	–	105.4	–	Kemenes, Forsberg & Melack (2007)
	Balbina Reservoir (2006)	–	161	–	109	Kemenes, Forsberg & Melack (2011)
				(0.5–287)		
						
Indonesia	Batanghari River (2009)	98 ± 0.7	–	20 ± 18	–	Wit et al. (2015)
	Indragiri River (2013)	236 ± 22	–	232 ± 61	–	
	Siak River (2013)	350 ± 22	–	320 ± 61	–	
						
Malaysia	Maludam River (2014)	319 ± 37	–	289 ± 155	–	Müller et al. (2015)
	Maludam River (2015)	343 ± 6	–	125 ± 136	–	
	Terengganu River (2018)	9.1	92	0.8	14	This study, median
		0.3–65	15–445	0.06–20	0.7–136	range

### Greenhouse gas emission dynamics in Terengganu River Estuary

The average CH_4_ concentrations in the estuary were significantly higher during high flow (9.2 ± 4.4 µM) than during low flow (3.6 ± 2.1 µM). Elevated CH_4_ levels observed during the high flow period in December 2017 were likely driven by substantial rainfall-induced urban runoff. Increased dissolved organic carbon during these events may have contributed to higher CH_4_ concentration in the estuary (Fig. 4). Previous studies have shown that river segments draining urban areas are important CH_4_ sources (Tang et al., 2021). CO_2_ and CH_4_ removal were observed from sites E7 to E15 during low flow periods, probably due to mixing with seawater in the lower estuary (Fig. 4).

Seasonally, the largest range of CO_2_ fluxes was observed during the dry season (September 2018), ranging from 70 to 350 mmol m^−2^ d^−1^ compared to the wet season (December 2017), which ranged from −11 to 110 mmol m^−2^ d^−1^ (Fig. 4). In contrast, average CH_4_ fluxes were higher in December 2017 (13.1 mmol m^−2^ d^−1^) than in September 2018 (4.1 mmol m^−2^ d^−1^) (*p* < 0.05). Overall, CO_2_ and CH_4_ efflux in TRE displayed a decreasing trend from the upper to the lower reaches (Fig. 4). The elevated CH_4_ emissions in the freshwater portion during December 2017 were likely due to inputs from nearby tributaries, stormwater discharge outlets, and non-point sources. Additionally, breakwater structure and land reclamation near the estuarine inlet may have altered water column mixing, potentially facilitating in *situ* GHG production (Fig. 4). This observation is consistent with Looman, Maher & Santos (2021), who reported increased localized outgassing due to altered watercourse hydrodynamics. Conversely, the decline in CO_2_ and CH_4_ fluxes in the lower estuary was likely driven by degassing from tidal oscillation and dilution with seawater.

The time-series CO_2_flux in the estuary varied primarily with tidal height rather than diurnal fluctuations (Fig. 5). During the spring tide, the maximum average CO_2_ flux reached 40 mmol m^−2^ d^−1^ during the daytime and 74 mmol m^−2^ d^−1^ at night. In contrast, during the neap tide, CO_2_ flux ranged from 3 to 53 mmol m^−2^ d^−1^, peaking at 51 mmol m^−2^ d^−1^ at 0400 h. The average CO_2_ flux during the daytime was 31 mmol m^−2^ d^−1^, while at night it was 17 mmol m^−2^ d^−1^.

Similarly, CH_4_ fluxes were strongly influenced by tidal conditions. During spring tides, the average CH_4_ flux reached 0.7 mmol m^−2^ d^−1^ during the ebb tide, significantly higher than the 0.008 mmol m^−2^ d^−1^ recorded during the flood tide. For neap tides, CH_4_ emissions showed a comparable amplitude to those during spring tides, ranging from 0.2 to 1.3 mmol m^−2^ d^−1^, with a peak of 1.3 mmol m^−2^ d^−1^at 1400 h. The average daytime emission was 0.9 mmol m^−2^ d^−1^, while nighttime average was 0.6 mmol m^−2^ d^−1^.

Although these differences suggest a possible diel component, tidal height appears to be the dominant factor controlling emissions. Higher tidal amplitudes during spring tides correspond to longer ebb tide duration (Mao et al., 2004), which enhances tidal damping and facilitates more efficient material exchange and seaward transport (Arueira et al., 2022; Geyer, Chant & Houghton, 2008; Siegle et al., 2009). During the ebb tide, as water levels in the estuary drop, the reduced water column allows trapped CO_2_ in the organic-rich sediment to be released more effectively.

Similarly, CH_4_ emission in estuaries strongly depends on tidal height variation, occurring predominantly at low tide and primarily in shallow water depth systems (Ferrón et al., 2007; Grunwald et al., 2007; Harley, 2013). Harley (2013) showed that a falling tide can partially replace the estuary water column with CH_4_-supersaturated freshwater from the upper estuary. Ferrón et al., (2007) and Grunwald et al. (2007) also observed that CH_4_ concentration increases as tidal amplitude and salinity decrease, attributing these changes to allochothonous inputs, urban effluent discharge, *in situ* production, or the drainage of CH_4_-rich pore water from tidal flats.

Table 4 compares CH_4_ diffusive flux in the studied estuary with other river estuaries worldwide. The CH_4_ flux in the estuary is comparable to the Jiulong River Estuary, China (Li et al., 2021), Adyar Estuary, India (Nirmal Rajkumar et al., 2008) and Scheldt Estuary, United Kingdom (Middelburg et al., 1996), spanning sub-tropical, tropical, and temperate regions. However, the CO_2_ diffusive flux in Terengganu River estuary exceeds the range reported for tropical river estuaries. It is an order of magnitude higher than the sub-tropical estuaries in China (Yu et al., 2013) and the USA (Jiang, Cai & Wang, 2008). High CH_4_-emitting estuaries in Table 4 share two key similarities: elevated suspended particulate matter levels and an external forcing mechanism that enhances CH_4_ production. These external factors include tidal induced resuspension (Li et al., 2021), prolonged water residence times due to reduced river discharge (Nirmal Rajkumar et al., 2008), freshwater inflow influced by sewage (Middelburg et al., 1996), and hydrodynamic alterations from breakwater and land reclamation (this study). Additonal contributing factors include pollution, monsoonal influences, and estuarine morphological changes (Borges, Abril & Bouillon, 2018; Gupta et al., 2009).

**Table 4:** CH_4_ and CO_2_ concentration and flux in estuaries around the world. Data represent the range (mean).

Study area	Country	Latitude	Dominant sources and processes	Measurement year	CH_4_	CO_2_	CH_4_	CO_2_	References
					Concentration range (µM)		Flux range (mmol m^−2^ d^−1^)		
Pulicat Lake Estuary	India	14°N	Lateral inputs	na	0.09–0.50 (0.24)		0.05–0.23		Shalini et al. (2006)
New River Estuary	USA	34°N	Lateral inputs	2016				−6.6–98.4	Van Dam, Edson & Tobias (2019)
Neuse River Estuary	USA	35°N	Lateral inputs	2016				−2.7–98.4	Van Dam, Edson & Tobias (2019)
Hugli Estuary	India	22°N	Lateral inputs	2014				(88.8)	Akhand et al. (2016)
Hooghly Estuary	India	21°N	Lateral inputs	1999				−2.8–84.4	Mukhopadhyay et al. (2002)
Betsibika Estuary (D)	Madagascar	18°S	Lateral inputs	2005		11–62.5		(9.1)	Ralison et al. (2008)
Guadalquivir Estuary	Spain	37°N	Tidal flats	2017	(0.05)	(39.2)	(0.12)	(60)	Sierra et al. (2020)
Guadiana Estuary	Spain	37°N	Tidal flats	2017	(0.05)	(24.3)	(0.11)	(40)	Sierra et al. (2020)
Tinto-Odiel Estuary	Spain	37°N	Tidal flats	2017	(0.04)	(23.7)	(0.07)	(11.7)	Sierra et al. (2020)
Matla Estuary	India	22°N	Tidal flats	2014				(6.3)	Akhand et al. (2016)
Rajang River Estuary (D)	Malaysia	2°N	Tidal flats	2016		(114)		15.9–68.2 (45.4)	Müller et al. (2019)
Rajang River Estuary (W)	Malaysia	2°N	Tidal flats	2016		(122)		15.9–54.5 (40.9)	Müller et al. (2019)
Lupar River Estuary (D)	Malaysia	1°N	Tidal flats	2013	0.01–0.04 (0.02)		<0		Müller et al. (2016)
Lupar River Estuary (W)	Malaysia	1°N	Tidal flats	2013	0–0.06 (0.02)		<0		Müller et al. (2016)
Saribas River Estuary (D)	Malaysia	1°N	Tidal flats	2013	0.01–0.06 (0.03)		<0		Müller et al. (2016)
Saribas River Estuary (W)	Malaysia	1°N	Tidal flats	2013	0.01–0.07 (0.02)		<0		Müller et al. (2016)
Lupar River Estuary (D)	Malaysia	1°N	Tidal flats	2014		(101)		(312)	Müller et al. (2016)
Lupar River Estuary (W)	Malaysia	1°N	Tidal flats	2014		(75.6)			Müller et al. (2016)
Saribas River Estuary (D)	Malaysia	1°N	Tidal flats	2014		(92.0)		(200)	Müller et al. (2016)
Saribas River Estuary (W)	Malaysia	1°N	Tidal flats	2014		(91.4)			Müller et al. (2016)
Furo do Meio in Caeté Estuary	Brazil	1°S	Tidal flats	2017			(0.86)	(174)	Call et al. (2018)
Altamaha Sound	USA	32°N	Tidal flats	2004				(69.3)	Jiang, Cai & Wang (2008)
Tagus Estuary	Portugal	38°N	Tidal dynamics	2007				24.5–65.5	Oliveira et al. (2018)
Changjiang River Estuary	China	29°N	Tidal dynamics	2006	0–0.09 (0.10)		(0.06)		Zhang et al. (2008)
Pearl River Estuary	China	26°N	Tidal dynamics	2003	0.02–2.98 (2.9)	28.2–110			Chen et al. (2008)
Jiulong River Estuary	China	25°N	Tidal dynamics	2018	0.06–1.7		0.19–18.6		Li et al. (2021)
Columbia River Estuary	USA	46°N	Landuse activity	2013	0.27–0.73 (0.45)				Pfeiffer-Herbert et al. (2019)
Oregon Estuary	USA	44°N	Landuse activity	1982			0–1.30		De Angelis Marie & Lilley Marvin (1987)
Mekong Delta	Vietnam	17°N	Landuse activity	2004	0-2.22	9.5–167	0.04–0.2	105–135	Borges, Abril & Bouillon (2018)
Scheldt Estuary	UK	51°N	Sewage	1991			0.14–189 (21.6)	17.5–227 (120)	Middelburg et al. (1996)
Hudson River Estuary	USA	42°N	Sewage	1991	0.05–0.94 (0.9)		(0.30)		DeAngelis & Scranton (1993)
Changjiang River Estuary	China	29°N	Sewage	2010		7.2–86		0–230 (61)	Yu et al. (2013)
Adyar Estuary (W)	India	13°N	Sewage	2001			(23.1)		Purvaja & Ramesh (2001)
Adyar Estuary (W)	India	13°N	Sewage	2004	0.03–5.62		0.04–11.0		Nirmal Rajkumar et al. (2008)
Cochin Estuary (D)	India	10°N	Sewage	2005		51.6–105		65–267	Gupta et al. (2009)
Brisbane River Estuary	Australia	27°S	Sewage	2012	0.08–0.31		0.25–2.2		Sturm et al. (2017)
Brisbane River Estuary	Australia	27°S	Sewage	2012	0.03–0.58 (0.5)		0.02–1.7		Musenze et al. (2013)
Godavari River Estuary (D)	India	19°N	Urbanization	2009		9–1339		80–150	Sarma et al. (2011)
Terengganu River Estuary (D)	Malaysia	5°N	Urbanization	2018	1.60–9.6 (4.0)	75–312 (212)	1.6–10.1	70–350 (233)	This study
Terengganu River Estuary (W)	Malaysia	5°N	Urbanization	2018	1.90–14.7 (5.7)	9–271 (135)	2.5–21.1	−11–391 (179)	This study
Caboolture River Estuary	Australia	27°S	Urbanization	2012				(78)	Jeffrey et al. (2018)

The total GHG emissions from the Terengganu River catchment are estimated at 572 Gg CO_2_-equivalent per year, with 85% CO_2_ and 15% CH_4_. This estimate applies to a global warming potential of 27 for CH_4_, based on a value from the IPCC Sixth Assessment Report (AR6). The catchment spans 386.6 km^2^, with the upper freshwater section (Kenyir Reservoir) covering 369 km^2^ and containing highly supersaturated dissolved GHG that influences the 61.5 km-long Terengganu River and its estuary. The river occupies ∼10 km^2^, while the estuary spans 7.6 km^2^. Average GHG flux from each section was multiplied by their respective surface areas, revealing that 94% of total emissions originated from the reservoir, 5.5% from the estuary, and only 0.5% from the river. Despite its smaller surface area than the river, the estuary contributes a disproportionately high share of emissions due to its higher organic matter content and reduced water turnover, exacerbated by the semi-enclosed breakwater that further elevated CH_4_ emissions during the wet season.

## Conclusions

**Figure 6:**
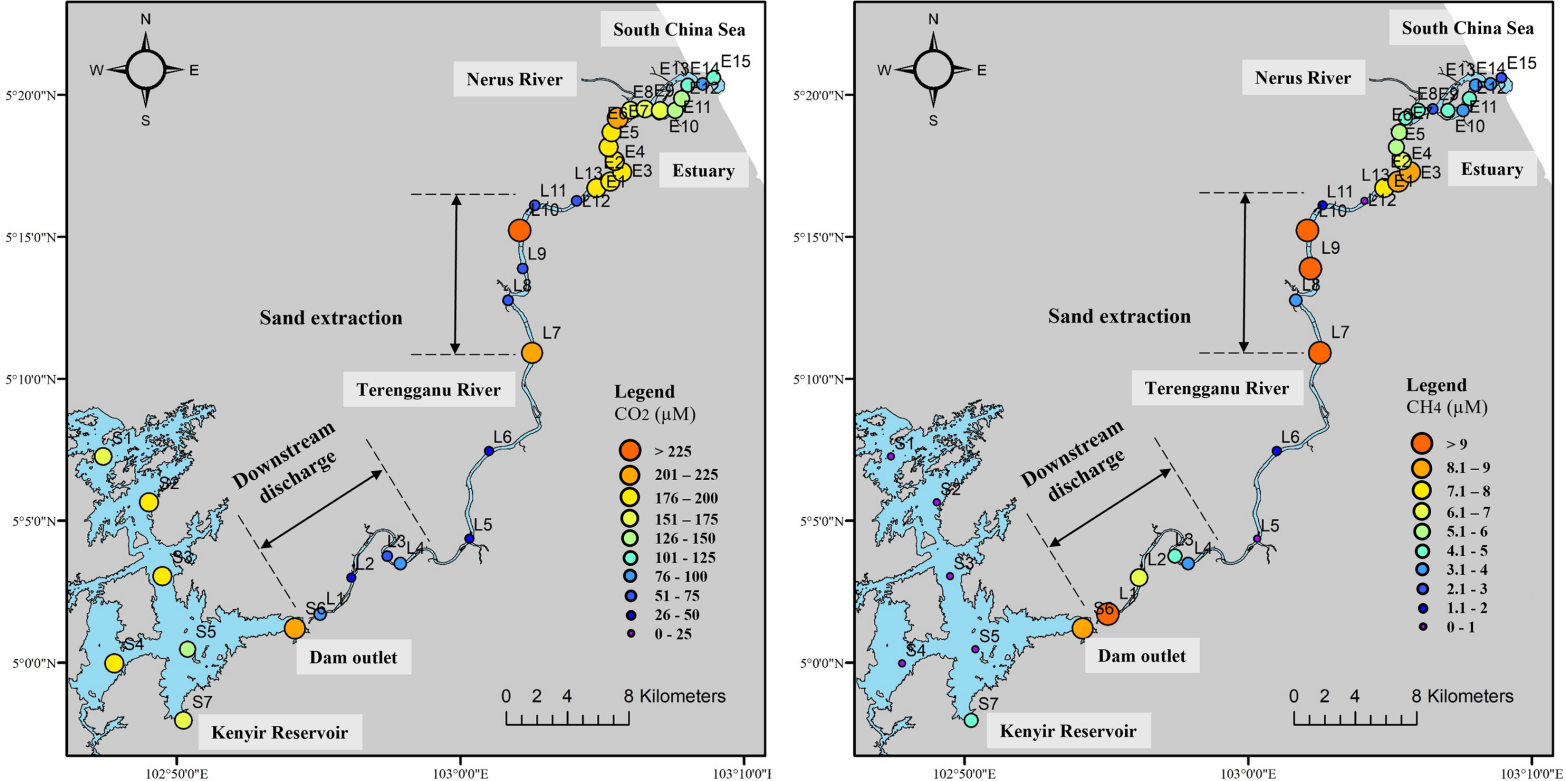
Greenhouse gases concentration in the entire Terengganu River system.

Human activities significantly shape the spatial distribution of CH_4_ and CO_2_ concentrations within the Terengganu River system (Fig. 6). High CH_4_ concentrations were observed downstream of the dam (S6), in the shallow littoral zone of the Kenyir Reservoir (S7), near dam discharge outlets (L1–L3), in river sections affected by sand mining (L7–L9), and in the upper estuary receiving urban drainage (E1–E4). Elevated CO_2_ levels were detected in the Kenyir Reservoir and upper estuary, particularly near sand extraction sites (L7) and at urban tributary outlets (E6).

This study highlights the critical role of hydropower reservoirs, such as Kenyir dam, as significant CO_2_ and CH_4_ emissions sources. The findings reveal substantial temporal and spatial variability in GHG emissions within a tropical river catchment, emphasizing how human activities, including river damming, sand extraction and uncontrolled urban drainage discharge, impact the river’s GHG dynamics. These insights underscore the need for further research to assess the long-term impacts of dam operations, urbanization, and other anthropogenic influences on GHG emissions in the Terengganu River system.

## Supplemental Information

10.7717/peerj.19929/supp-1 Supplemental Information 1 Raw data

10.7717/peerj.19929/supp-2 Supplemental Information 2 Summary of sampling campaign and research activities in this study

10.7717/peerj.19929/supp-3 Supplemental Information 3 Coefficient for least squares third-order polynomial fits of Schmidt number versus temperature for seawater (35%) and fresh water for temperature ranging from 0 to 30 ° C (Wanninkhof (1992)

10.7717/peerj.19929/supp-4 Supplemental Information 4 Literature *k*_600_ equation for both lake and estuary * The codes represent the authors and publication years of the gas transfer velocity parameterizations used.

10.7717/peerj.19929/supp-5 Supplemental Information 5 Basic characteristics of Kenyir Reservoir, Terengganu River and Estuary and Wind Data * Historical local wind data from global models as described in Zippendenig (2024)

10.7717/peerj.19929/supp-6 Supplemental Information 6 Schematic diagram of the ebullition flux chamber (1) 70mm PVC pipe, (2) inverted wok, (3) brass nozzle, (4 and 5) 6mm tubing, (6) outlet with 21G needle and 3-way valve, (7) inlet with 21G needle and 3-way valve, (8) 60 mL butyl septa serum bottle, (9) cable tie, (10) float, (11) bolt and nut.

10.7717/peerj.19929/supp-7 Supplemental Information 7 Spatial and temporal water quality parameters in Kenyir reservoir, Terengganu River and Estuary

10.7717/peerj.19929/supp-8 Supplemental Information 8 Comparison of CO_2_ and CH_4_ concentrations (µM) between stations Statistical comparison using the Kruskal–Wallis test shows significant differences in CO_2_and CH_4_ concentrations among stations (*p* < 0.05), while no significant difference was observed for CO_2_and CH_4_ fluxes (*p* > 0.05).

10.7717/peerj.19929/supp-9 Supplemental Information 9 Temporal dissolved oxygen and CH_4_ concentration profiles in Kenyir reservoir

## Additional Information and Declarations

### Data Availability

The following information was supplied regarding data availability: Raw data is available in the Supplemental Files.
