# Supplementary material for: An assessment of CO2 and CH4 emissions in a tropical river: from the Kenyir Reservoir to the estuary
Source: PeerJ. 2025 Sep 3;13:e19929. doi: 10.7717/peerj.19929 (PMC12422260; doi:10.7717/peerj.19929)
Supplement: Supplemental Information 2 [file peerj-13-19929-s002.docx]

| Study Area | Season | Sampling | Period | Performed Activities |
| --- | --- | --- | --- | --- |
| **Kenyir Reservoir** | | | | |
|  | Wet | First | Feb-18 | discrete-depth sampling |
|  | Dry | Second | Mar-18 | discrete-depth sampling |
|  | Dry | Third | May-18 | discrete-depth sampling |
|  | Dry | Fourth | Jul-18 | discrete-depth sampling |
|  | Wet | Fifth | Nov-18 | discrete-depth sampling |
| **Terengganu River (Immediate downstream)** | | | | |
| High discharge (>150 m^3^ s^-1^) | | First | Mar-18 | Longitudinal sampling |
| High discharge (>150 m^3^ s^-1^) | | Second | Apr-18 | Longitudinal sampling |
| Low discharge (<150 m^3^ s^-1^) | | Third | Apr-19 | Longitudinal sampling |
| **Terengganu River Estuary** | | | | |
|  | Wet | First | Dec-17 | Longitudinal sampling |
|  | Dry | Second | Sep-18 | Longitudinal sampling |
|  | Wet | Third | Dec-18 | Longitudinal sampling |
|  | Spring | Fourth | Apr-19 | 24 hrs time series |
|  | Neap | Fifth | Aug-19 | 24 hrs time series |
