# Supplementary material for: An assessment of CO2 and CH4 emissions in a tropical river: from the Kenyir Reservoir to the estuary
Source: PeerJ. 2025 Sep 3;13:e19929. doi: 10.7717/peerj.19929 (PMC12422260; doi:10.7717/peerj.19929)
Supplement: Supplemental Information 3 [file peerj-13-19929-s003.docx]

|  |
| --- |
| \| Gas \| A \| B \| C \| D \| \| --- \| --- \| --- \| --- \| --- \| \| Seawater \| \| \| \| \| \| CO_2_ \| 2073.1 \| 125.62 \| 3.6276 \| 0.043219 \| \| CH_4_ \| 2039.2 \| 120.31 \| 3.4209 \| 0.040437 \| \| Fresh Water \| \| \| \| \| \| CO_2_ \| 1911.1 \| 118.11 \| 3.4527 \| 0.04132 \| \| CH_4_ \| 1897.8 \| 114.28 \| 3.2902 \| 0.039061 \| |
