# Supplementary material for: An assessment of CO2 and CH4 emissions in a tropical river: from the Kenyir Reservoir to the estuary
Source: PeerJ. 2025 Sep 3;13:e19929. doi: 10.7717/peerj.19929 (PMC12422260; doi:10.7717/peerj.19929)
Supplement: Supplemental Information 4 — * The codes represent the authors and publication years of the gas transfer velocity parameterizations used. [file peerj-13-19929-s004.docx]

|  |
| --- |
| \| Lake \| Reference \| \| --- \| --- \| \| W92 $\boldsymbol{k}_{\boldsymbol{600}}\boldsymbol{= 0.31}\boldsymbol{u}_{\boldsymbol{10}}^{\boldsymbol{2}}$ \| Wanninkhof (1992) \| \| M95 $\boldsymbol{k}_{\boldsymbol{600}}\boldsymbol{= 0.45}\boldsymbol{u}_{\boldsymbol{10}}^{\boldsymbol{1.6}}$ \| MacIntyre and Melack (1995) \| \| C&W03 $\boldsymbol{k}_{\boldsymbol{600}}\boldsymbol{= 0.168+0.228}\boldsymbol{u}_{\boldsymbol{10}}^{\boldsymbol{2.2}}$ \| Crusius and Wanninkhof (2003) \| \| C10 $\boldsymbol{k}_{\boldsymbol{600}}\boldsymbol{= 0.497+0.0064}\boldsymbol{u}_{\boldsymbol{10}}^{\boldsymbol{1.8}}$ \| Cole *et al.* (2010) \| \| Estuary \| **Reference** \| \| C95 $\boldsymbol{k}_{\boldsymbol{600}}\boldsymbol{=2.0 \times0.24}\boldsymbol{u}_{\boldsymbol{10}}$ \| Clark *et al.* (1995) \| \| R&C01 $\boldsymbol{k}_{\boldsymbol{600}}\boldsymbol{=1.91 \times}\boldsymbol{exp}^{\boldsymbol{0.35}_{\boldsymbol{U}_{\boldsymbol{10}}}}$ \| Raymond and Cole (2001) \| \| B04 $\boldsymbol{k}_{\boldsymbol{600}}\boldsymbol{=4.045+ 2.580 \times}\boldsymbol{u}_{\boldsymbol{10}}$ \| Borges *et al.* (2004) \| \| W09$\boldsymbol{k}_{\boldsymbol{660}}\boldsymbol{=3+ 0.1}\boldsymbol{u}_{\boldsymbol{10}}\boldsymbol{+ 0.064}\boldsymbol{u}_{\boldsymbol{10}}^{\boldsymbol{2}}\boldsymbol{+ 0.01}\boldsymbol{u}_{\boldsymbol{10}}^{\boldsymbol{3}}$ \| Wanninkhof *et al.* (2009) \| |

Reference

Bastviken D, Cole J, Pace M, Tranvik L. 2004. Methane emissions from lakes: dependence of lake characteristics, two regional assessments, and a global estimate. Global Biogeochemical Cycles 18(4) DOI 10.1029/2004GB002238.

Clark J, Schlosser P, Simpson H, Stute M, Wanninkhof R, Ho D. 1995. Relationship between gas transfer velocities and wind speeds in the tidal Hudson River determined by the dual tracer technique. In B. Jahne & E.C. Monohan (Eds.), Air-Water Gas Transfer (785-800). AEON Verlag & Studio Hanau.

Cole JJ, Bade DL, Bastviken D, Pace ML, Van De Bogert M. 2010. Multiple approaches to estimating air-water gas exchange in small lakes. Limnology and Oceanography Methods 8(6):285 293 DOI 10.4319/lom.2010.8.285.

Crusius J, Wanninkhof R. 2003. Gas transfer velocities measured at low wind speed over a lake. Limnology and Oceanography 48(3):1010 1017 DOI10.4319/lo.2003.48.3.1010.

MacIntyre S, Melack JM. 1995. Vertical and horizontal transport in lakes: linking littoral, benthic, and pelagic habitats. Journal of the North American Benthological Society 14(4):599 615 DOI 10.2307/1467544.

Raymond PA, Cole JJ. 2001. Gas exchange in rivers and estuaries: choosing a gas transfer velocity. Estuaries 24(2):312 DOI 10.2307/1352954.

Wanninkhof R. 1992. Relationship between wind speed and gas exchange over the ocean. Journal of Geophysical Research 97(C5):7373 7382 DOI 10.1029/92JC00188.

Wanninkhof R, Asher WE, Ho DT, Sweeney C, McGillis WR. 2009. Advances in quantifying air-sea gas exchange and environmental forcing. Annual Review of Marine Science 1(1):213 244 DOI 10.1146/annurev.marine.010908.163742.
