# Supplementary material for: An assessment of CO2 and CH4 emissions in a tropical river: from the Kenyir Reservoir to the estuary
Source: PeerJ. 2025 Sep 3;13:e19929. doi: 10.7717/peerj.19929 (PMC12422260; doi:10.7717/peerj.19929)
Supplement: Supplemental Information 5 — * Historical local wind data from global models as described in Zippendenig (2024) [file peerj-13-19929-s005.docx]

| **General Hydropower Dam** | Country | Malaysia | |
| --- | --- | --- | --- |
|  | River | Previously Kenyir River | |
|  | Latitude | 04°47’ N to 05°15’ N | |
|  | Longitude | 102°32’ E to 102°55’ E | |
|  | Year of impoundment | 1985 | |
|  | Installed Capacity | 400 MW | |
|  | Annual Power Production | 1600 GWh | |
|  | | | |
| **Meteorology** |  | **Mean** | **Range** |
|  | Monthly average wind speed (m/s) | 1.27 | 0.96-1.78 |
|  | Annual Precipitation (mm) | 2714.5 | 0-3200 |
|  | Air temperature (°C) | 26.7 | 26.5-32 |
|  | | | |
| **Kenyir Reservoir** | Surface at full water level (km^2^) | 369 | - |
|  | Catchment Area (km^2^) | 1260 | - |
|  | Drawdown level (m) | 10 | 5-15 |
|  | Depth / Maximum Depth | 145m | - |
|  | Surface water temperature (°C) | 29.7 | 28.1-31.7 |
|  | Thermocline depth | 25 | 15-40 |
|  | | | |
| **Terengganu River** | Total Length (km) | 61.5 | - |
|  | Surface area (km^2^) | 10.0 | - |
|  | Surface water temperature (°C) | 28.9 | 26.7-31 |
|  | Annual Water discharge (m^3^ s^-1^) | 175.7 | 147-224 |
|  | | | |
| **Terengganu River Estuary** | Estimated length (km) | 9 | - |
|  | Surface area (km_2_) | 7.6 | - |
|  | Surface water temperature (°C) | 27.7 | 26.2-31.6 |
|  | Water depth (m) | 4.7 | 5-12 |

| **Month** | ***Average of wind speed 10m**  **(m/s)** | **Min of wind speed 10m (m/s)** | **Max of wind speed 10m (m/s)** |
| --- | --- | --- | --- |
| Jan-18 | 1.39 | 0.00 | 5.24 |
| Feb-18 | 1.78 | 0.00 | 4.92 |
| Mar-18 | 1.55 | 0.00 | 3.94 |
| Apr-18 | 1.44 | 0.14 | 3.94 |
| May-18 | 1.13 | 0.00 | 3.92 |
| Jun-18 | 1.04 | 0.00 | 3.55 |
| Jul-18 | 0.96 | 0.00 | 2.62 |
| Aug-18 | 1.06 | 0.00 | 3.31 |
| Sep-18 | 1.07 | 0.00 | 3.33 |
| Oct-18 | 1.21 | 0.10 | 4.10 |
| Nov-18 | 1.27 | 0.00 | 4.10 |
| Dec-18 | 1.40 | 0.10 | 4.32 |
