# Supplementary figures and images for: An assessment of CO2 and CH4 emissions in a tropical river: from the Kenyir Reservoir to the estuary
Source: PeerJ. 2025 Sep 3;13:e19929. doi: 10.7717/peerj.19929 (PMC12422260; doi:10.7717/peerj.19929)

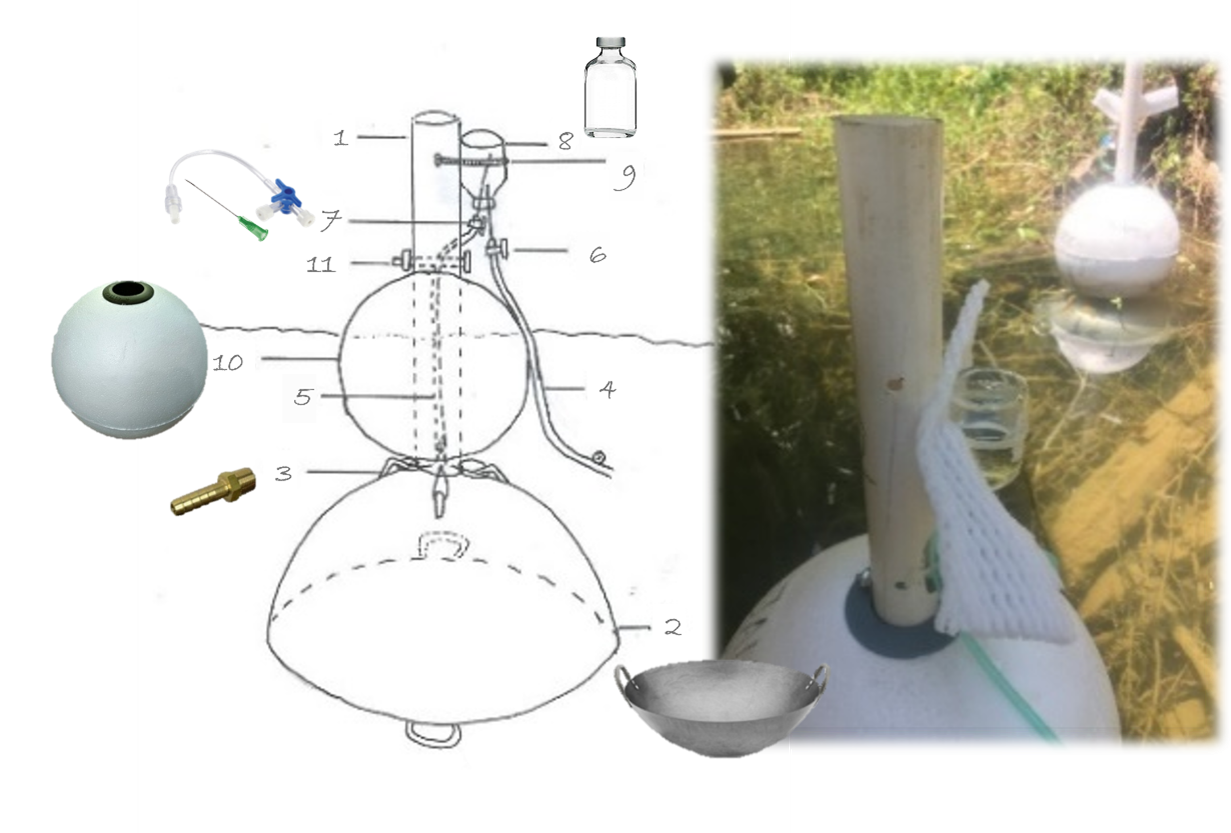

Supplement: Supplemental Information 6 — (1) 70mm PVC pipe, (2) inverted wok, (3) brass nozzle, (4 and 5) 6mm tubing, (6) outlet with 21G needle and 3-way valve, (7) inlet with 21G needle and 3-way valve, (8) 60 mL butyl septa serum bottle, (9) cable tie, (10) float, (11) bolt and nut. [file peerj-13-19929-s006.png]

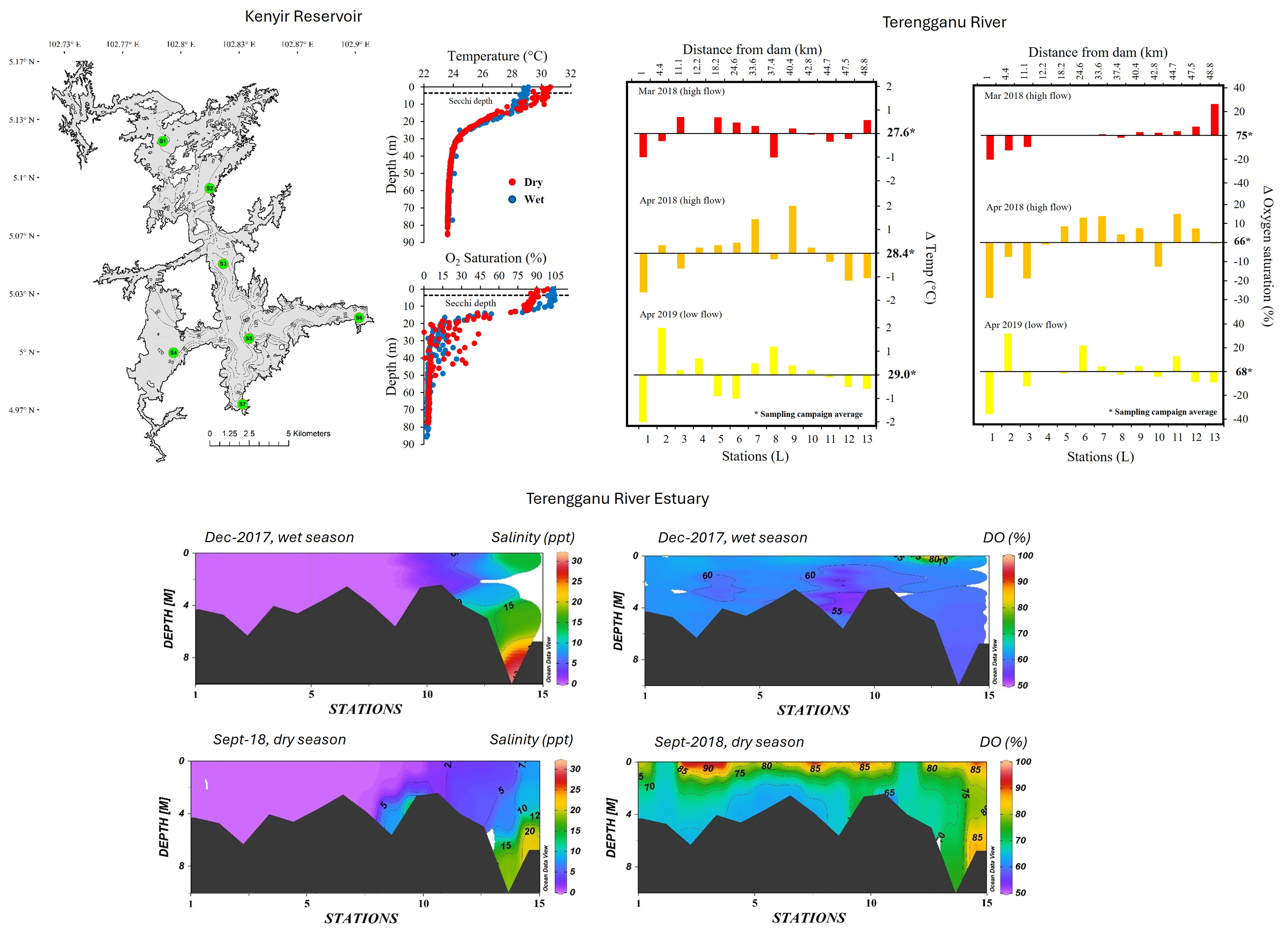

Supplement: Supplemental Information 7 [file peerj-13-19929-s007.png]

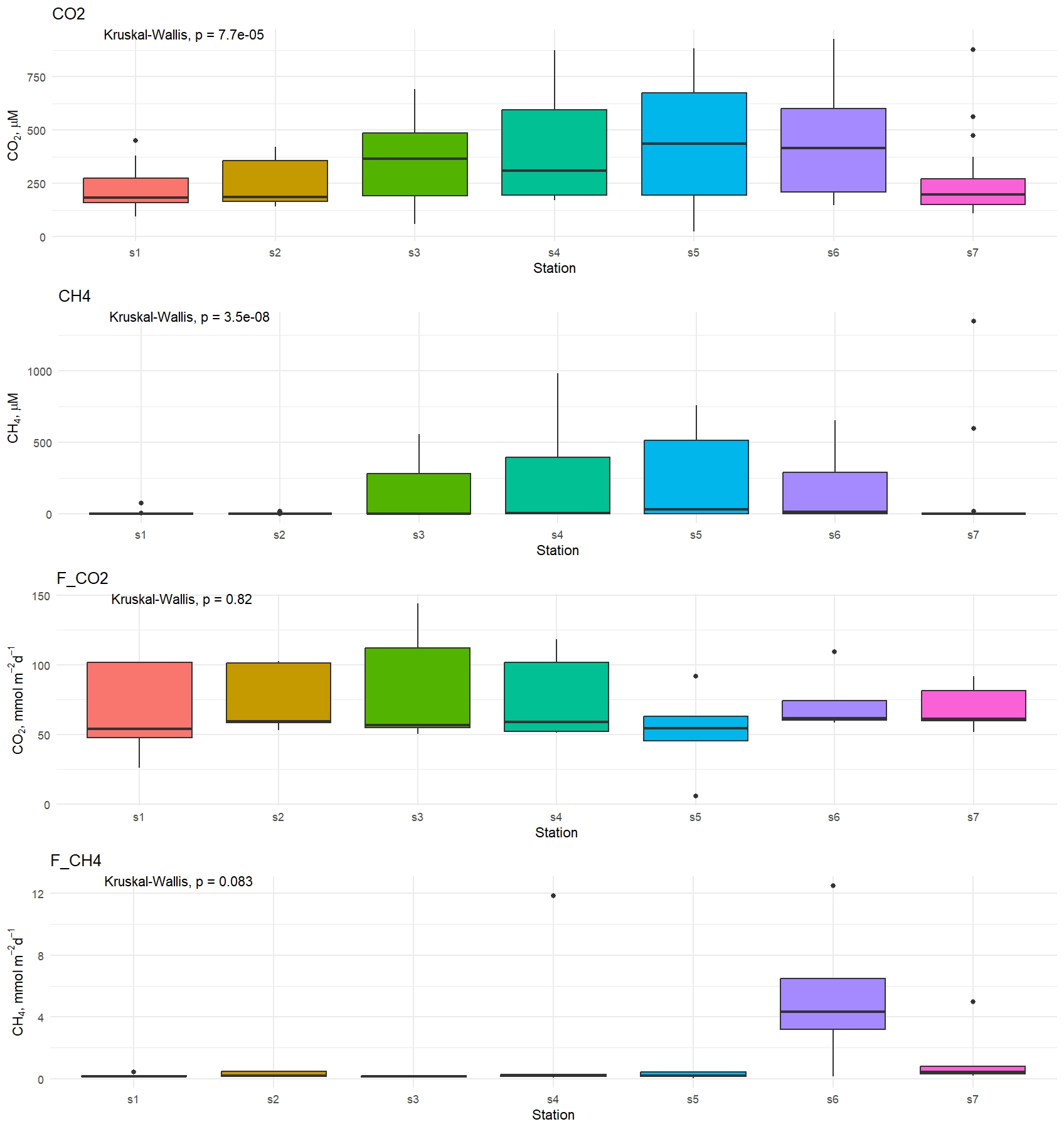

Supplement: Supplemental Information 8 — Statistical comparison using the Kruskal–Wallis test shows significant differences in CO2and CH4 concentrations among stations (p < 0.05), while no significant difference was observed for CO2and CH4 fluxes (p > 0.05). [file peerj-13-19929-s008.jpeg]

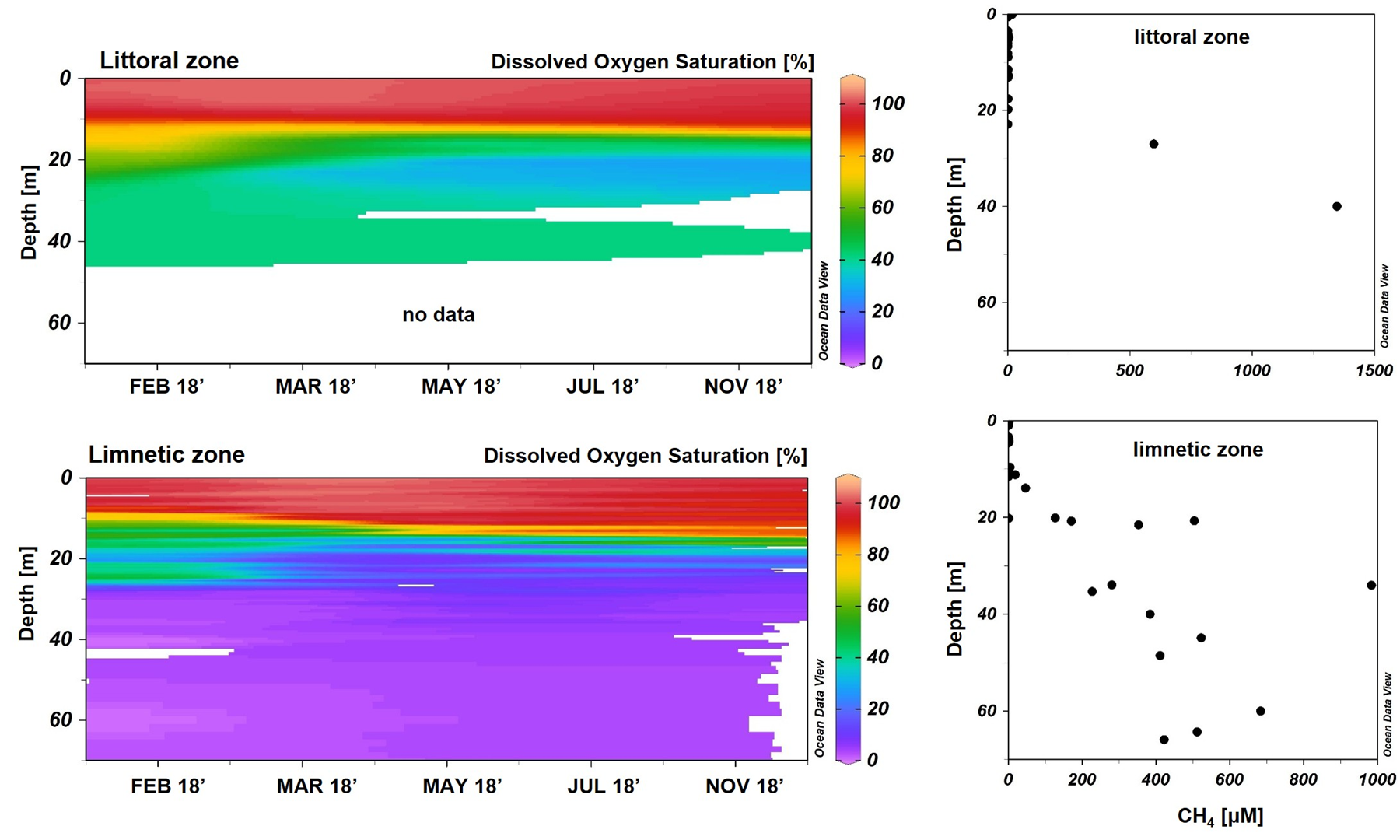

Supplement: Supplemental Information 9 [file peerj-13-19929-s009.png]
